# Defective airway intraflagellar transport underlies a combined motile and primary ciliopathy syndrome caused by *IFT74* mutations

**DOI:** 10.1093/hmg/ddad132

**Published:** 2023-08-09

**Authors:** Mahmoud R Fassad, Nisreen Rumman, Katrin Junger, Mitali P Patel, James Thompson, Patricia Goggin, Marius Ueffing, Tina Beyer, Karsten Boldt, Jane S Lucas, Hannah M Mitchison

**Affiliations:** Genetics and Genomic Medicine Research and Teaching Department, University College London, UCL Great Ormond Street Institute of Child Health, 30 Guilford Street, London WC1N 1EH, United Kingdom; Department of Human Genetics, Medical Research Institute, Alexandria University, 22 El-Guish Road, El-Shatby, Alexandria 21526, Egypt; Department of Pediatrics, Faculty of Medicine, Makassed Hospital and Al-Quds University, East Jerusalem 91220, Palestine; Section of Pulmonary, Critical Care and Sleep Medicine, Department of Internal Medicine, Yale University School of Medicine, 300 Cedar St #441, New Haven, CT 06520, United States; Institute for Ophthalmic Research, Eberhard Karl University of Tübingen, Elfreide-Alhorn-Strasse 5-7, Tübingen 72076, Germany; Genetics and Genomic Medicine Research and Teaching Department, University College London, UCL Great Ormond Street Institute of Child Health, 30 Guilford Street, London WC1N 1EH, United Kingdom; MRC Prion Unit at UCL, Institute of Prion Diseases, University College London, 33 Cleveland Street, London W1W 7FF, United Kingdom; Primary Ciliary Dyskinesia Centre, NIHR Biomedical Research Centre, University Hospital Southampton NHS Foundation Trust, Southampton SO16 6YD, United Kingdom; School of Clinical and Experimental Sciences, University of Southampton Faculty of Medicine, University Road, Southampton SO17 1BJ, United Kingdom; Biomedical Imaging Unit, University of Southampton Faculty of Medicine, University Road, Southampton SO17 1BJ, United Kingdom; Primary Ciliary Dyskinesia Centre, NIHR Biomedical Research Centre, University Hospital Southampton NHS Foundation Trust, Southampton SO16 6YD, United Kingdom; Biomedical Imaging Unit, University of Southampton Faculty of Medicine, University Road, Southampton SO17 1BJ, United Kingdom; Institute for Ophthalmic Research, Eberhard Karl University of Tübingen, Elfreide-Alhorn-Strasse 5-7, Tübingen 72076, Germany; Institute for Ophthalmic Research, Eberhard Karl University of Tübingen, Elfreide-Alhorn-Strasse 5-7, Tübingen 72076, Germany; Institute for Ophthalmic Research, Eberhard Karl University of Tübingen, Elfreide-Alhorn-Strasse 5-7, Tübingen 72076, Germany; Primary Ciliary Dyskinesia Centre, NIHR Biomedical Research Centre, University Hospital Southampton NHS Foundation Trust, Southampton SO16 6YD, United Kingdom; School of Clinical and Experimental Sciences, University of Southampton Faculty of Medicine, University Road, Southampton SO17 1BJ, United Kingdom; Genetics and Genomic Medicine Research and Teaching Department, University College London, UCL Great Ormond Street Institute of Child Health, 30 Guilford Street, London WC1N 1EH, United Kingdom

**Keywords:** IFT74, ciliopathies, primary ciliary dyskinesia, intraflagellar transport, IFT-B

## Abstract

Ciliopathies are inherited disorders caused by defective cilia. Mutations affecting motile cilia usually cause the chronic muco-obstructive sinopulmonary disease primary ciliary dyskinesia (PCD) and are associated with laterality defects, while a broad spectrum of early developmental as well as degenerative syndromes arise from mutations affecting signalling of primary (non-motile) cilia. Cilia assembly and functioning requires intraflagellar transport (IFT) of cargos assisted by IFT-B and IFT-A adaptor complexes. Within IFT-B, the N-termini of partner proteins IFT74 and IFT81 govern tubulin transport to build the ciliary microtubular cytoskeleton. We detected a homozygous 3-kb intragenic *IFT74* deletion removing the exon 2 initiation codon and 40 N-terminal amino acids in two affected siblings. Both had clinical features of PCD with bronchiectasis, but no laterality defects. They also had retinal dysplasia and abnormal bone growth, with a narrowed thorax and short ribs, shortened long bones and digits, and abnormal skull shape. This resembles short-rib thoracic dysplasia, a skeletal ciliopathy previously linked to IFT defects in primary cilia, not motile cilia. Ciliated nasal epithelial cells collected from affected individuals had reduced numbers of shortened motile cilia with disarranged microtubules, some misorientation of the basal feet, and disrupted cilia structural and IFT protein distributions. No full-length IFT74 was expressed, only truncated forms that were consistent with N-terminal deletion and inframe translation from downstream initiation codons. In affinity purification mass spectrometry, exon 2-deleted IFT74 initiated from the nearest inframe downstream methionine 41 still interacts as part of the IFT-B complex, but only with reduced interaction levels and not with all its usual IFT-B partners. We propose that this is a hypomorphic mutation with some residual protein function retained, which gives rise to a primary skeletal ciliopathy combined with defective motile cilia and PCD.

## Introduction

Ciliopathies are a large group of Mendelian disorders characterized by defective structure and function of cilia, hairlike organelles that extend out from the cell surface (1–4). In humans, these are present either as primary, non-motile cilia located on most cell types that are responsible for cell signal transduction or as motile cilia present on specialized epithelia, which are responsible for particle flow or clearance of fluids. These different cilia have distinctive, highly conserved internal axonemal architectures, consisting of nine peripheral microtubules either with (9 + 2, motile cilia) or without (9 + 0, primary cilia or motile nodal cilia) a central microtubule pair, along with additional motility machinery attached to the microtubules of the motile cilia (e.g. dynein arms) (5–7).

Our understanding of cilia-related genes is evolving and currently, mutations in at least 300 different genes are recognized to cause different primary and motile cilia disorders (5,8–10). Consistent with the complex distribution and roles of cilia, the human ciliopathies are collectively a heterogeneous disease group affecting multiple systems in the body, spanning skeletal anomalies, craniofacial defects, cystic kidneys, blindness, obesity, respiratory, laterality and fertility problems, and other presentations.

Ciliopathy gene mutations often affect components of the highly conserved intraflagellar transport system of cilia (IFT). IFT is required for regulated protein cargo transport in and out of the cilium since cilia lack protein synthesis machinery and their components need to be imported from the cell body. IFT is therefore essential for the assembly, growth, and proper functioning of both primary and motile cilia (4,11,12). IFT traffics and distributes the protein cargos required for ciliogenesis, cilia structure, motility (tubulin, outer and inner dynein arms), and signal transduction (e.g., Hedgehog signalling). Cargos are imported into cilia and moved up and down the axoneme microtubules by large IFT ‘trains’, which consist of IFT motor proteins attached to the cargos through multi-protein adaptor complexes called IFT-A and IFT-B. As extensively reviewed (4,13–15), IFT-B, which governs anterograde (base-to-tip) transport, contains 16 proteins as a 10-protein core split into B1–1 (IFT25, IFT27, IFT74, IFT81, IFT22) and B1–2 (IFT56, IFT46, IFT52, IFT88, IFT70) subcomplexes, plus a 6-protein peripheral (B2: IFT57, IFT80, IFT20, IFT172, CLUAP1/IFT38, TRAF3IP1/IFT54) subcomplex. The 6-protein IFT-A complex (IFT144, IFT140, IFT139, IFT122, IFT121, IFT43) governs retrograde (tip-to-base) ciliary transport.

IFT gene mutations are a prominent cause of human primary ciliopathies linked to dysfunction of primary cilia. While mutations of the IFT-A complex are often associated with abnormal cilia architecture and bulging ciliary tips with accumulated IFT particles, mutations of components of the IFT-B complex typically block cilia assembly, leading to shortened or absent cilia, in humans and model organisms (16). IFT-B mutations cause several different ciliopathy diseases with varied disease features often towards the severe and lethal end of the ciliopathies phenotypic spectrum, including skeletal and retinal involvement (17–23). The currently recognized spectrum of IFT-B disorders is complex and covers multiple ciliopathy phenotypes, with skeletal involvement including asphyxiating thoracic dystrophy (ATD, Jeune syndrome) and short-rib thoracic dysplasias (short rib polydactylies) that occur with/or without polydactyly and kidney disease (fibrosis, nephronophthisis, Mainzer–Saldino syndrome), along with variable cardiac, hepatic (cholestasis, fibrosis), or retinal involvement (*IFT52, TRAF3IP1/IFT54, IFT56, IFT74, IFT80, IFT81, IFT172* mutations). There can also be neurological (developmental delay, cerebellar atrophy, molar tooth sign), limb, and craniofacial anomalies that overlap with features of Joubert, oral–facial–digital, and Pallister–Hall syndromes (*CLUAP1/IFT38, IFT56, IFT74, IFT81*), in addition to Bardet–Biedl syndrome, i.e. developmental delay, retinal degeneration, polydactyly, obesity, and hypogonadism (*IFT27, IFT74, TRAF3IP1/IFT54, IFT172*).

Defects of motile cilia have not been significantly linked to IFT gene mutations. Although IFT was first described in the motile cilia (flagella) of the model organism *Chlamydomonas reinhardtii* (24), motile cilia defects are mostly not evident in patients with IFT mutations and respiratory symptoms are usually attributed secondarily to chest dysplasia, short ribs, and lung restriction. Lethal respiratory failure is usually linked to perinatal lethal short-rib polydactyly ciliopathy syndromes, which can be caused by either IFT-B or IFT-A gene mutations (18–20,25–27).

The major disorder arising from motile cilia dysfunction is primary ciliary dyskinesia (PCD), which can be caused by mutations in at least 50 different genes. PCD mutations often affect genes involved in the assembly, structure, spacing, and regulation of cilia motors and other cilia motility proteins that regulate cilia beating and waveform; related sperm tail dysfunction also occurs (9,28). Reflecting the presence of cilia in the airways, PCD patients are usually diagnosed in respiratory or ENT clinics based on respiratory distress at birth, daily wet cough, recurrent chest infections, chronic rhinitis and sinusitis, otitis media, and hearing loss, with progression into bronchiectasis and irreversible lung damage (29,30). Defective cilia motility also occurs in the embryonic node, brain ventricles and fallopian tubes. As a result, half of PCD cases have laterality defects, commonly situs inversus, with a proportion of related heterotaxias and heart abnormalities. Furthermore, certain PCD gene defects are linked to hydrocephalus. In addition, subfertility affects both sexes and males with PCD often have defective airway cilia accompanied by infertility with malformed, short, and immotile sperm tails (31).

There are rare forms of human motile ciliopathy/PCD with syndromic features (developmental delay, retinal dysfunction) in addition to airway disease, which share mutations in genes more commonly linked to primary ciliopathies (32–35). However, few formal associations of gene mutations causing skeletal involvement in human PCD have been reported. Here, we report PCD and motile cilia defects associated with mutations in *IFT74*, a component of the anterograde IFT-B complex, and show the consequences of an IFT74 inframe N-terminal deletion on its interactions with other IFT-B components.

## Materials and methods

### Patients and genetic analysis

The study was ethically approved by the London Bloomsbury Research Ethics Committee (08/H0713/82) and the ethics committee at the Faculty of Medicine, Alexandria University. Informed consent was obtained from the parents. Clinical analysis and diagnostics of patients followed the clinical guidelines as described previously (36). Molecular genetics analysis was done using targeted next-generation sequencing (NGS) as described before (36,37) with filtering using the gene panel listed in Supplementary Material, Table S1. The impact on splicing was predicted using *in silico* tools, e.g. Alamut v2.10 and Human Splicing Finder v3.1. The prioritized variants were confirmed in the two affected siblings and segregated within the family using Sanger sequencing. PCR primers available on request, genomic deletion breakpoint primers are listed in Supplementary Material, Table S2.

### Immunofluorescence and electron microscopy

Multiciliated epithelial cells were obtained by nasal brushing biopsies. The brushing samples of patients and normal controls were dissociated in serum-free medium (M199) (Sigma-Aldrich) and applied to glass slides. The slides were left to dry for use in immunofluorescence staining of ciliated cells from patients and controls, as previously described (38). All antibodies and dilutions used are listed in Supplementary Material, Table S3. For TEM, nasal brushing biopsies were fixed in 4% glutaraldehyde followed by processing and quantification as previously described (36).

### Real-time PCR and quantitative PCR

Total RNA was isolated from nasal brushing samples using the Trizol reagent (Qiagen Inc.). 0.5–2 μg of RNA was used for cDNA synthesis using the High Capacity RNA-to-cDNA Kit (ThermoFisher Scientific) according to the manufacturers’ protocol. Primers used for RT-PCR and RT-qPCR were designed using Primer-Blast with a chosen PCR product size of 80–150 bp. *GAPDH* was used as an internal control (primers listed in Supplementary Material, Table S2). Real-time PCR was done using the CFX96 Touch™ Real-Time PCR Detection System (Bio-Rad Labs Inc., CA, USA) and the iQ SYBR Green Supermix (Bio-Rad) with each PCR reaction done in triplicate, 100 ng cDNA mixed with 10 μl iQ SYBR Green Supermix and 150 nM of each forward and reverse primers, to a final volume of 20 μl. The thermal cycling protocol was set up for a two-step PCR reaction starting with 3 min at 95 °C for polymerase activation and DNA denaturation followed by 40 cycles of 15-s denaturation at 95 °C and 30-s annealing/extension at 60 °C. Melting curve analysis was done at 55–95 °C (in 0.5 °C increments) for 30 s, to confirm the specificity of the primers used. Standard curves were generated separately for each gene using serial cDNA dilutions of pooled samples to assess the efficiency of PCR reactions. Data were analysed using Bio-Rad CFX Manager 3.1 software (Bio-Rad Labs Inc., CA, USA). Relative quantification of gene expression was performed using the delta–delta Ct (2-ΔΔCt) method, where target gene expression was normalized to an endogenous housekeeping gene (*GAPDH*) expression and to a calibrator sample (control) as previously described (39).

### Western blotting

Protein lysates were prepared from nasal brushing biopsies collected in RNAlater (Sigma-Aldrich). RNAlater was diluted with equal 1x PBS and centrifuged to pellet the cells. NP-40 lysis buffer supplemented with protease inhibitor cocktail was added, and the pellet homogenized. The suspension was incubated at 4 °C with agitation then the lysate was centrifuged and the supernatant saved. Quantification of the protein yield was done using a Pierce BCA Protein Assay Kit (ThermoFisher Scientific) and samples run on western blots as previously described (38).

### HEK293 cell transfection

HEK293 cells were transfected with Strep-FLAG-tagged IFT74 constructs and a negative control (RAF1 FLAG-plasmid) in six replicate experiments. One day prior to transfection, HEK293T cells were seeded at a density of 0.5 × 106 cells/well in six-well plates to reach 70–90% confluency at the time of transfection. Lipofectamine 3000 or 2000 (ThermoFisher) was diluted in Opti-MEM serum-free medium and used for application to the cells according to the manufacturer’s protocol. 2.5 μg of DNA was used for transfection per well in a six-well plate, and the cells were incubated at 37 °C and 5% CO_2_ for 48 h. The amount of DNA and lipofectamine used were scaled up/down according to the surface area of the culture plate/dish/flask.

### Affinity purification and mass spectrometry

HEK293 transfections were followed by affinity purification of Strep-FLAG fusion proteins then mass spectrometry and LFQ of the eluates, using methods previously described (40). For identification and quantification of proteins, the raw spectra were searched against the human SwissProt databases (03/2018) using MaxQuant software (version 1.5.0.3) with a minimum ratio count of 2. The first search peptide tolerance was set to 20, the main search peptide tolerance to 4.5 ppm, and the ‘re-quantify’ option was selected. Contaminants were detected using the MaxQuant contaminant search. A minimum peptide number of two peptides and a minimum length of seven amino acids was tolerated. An FDR of protein and peptides was set at 1% with unique and razor peptides used for quantification. For statistical analysis, the free Perseus software package (version 1.5.3.3) was used. Proteins with a quantitative value in less than 50% of the samples of at least one group were removed. Missing values were imputed from the normal distribution with a width of 0.8 and a down shift of 1.3. To test for differences between groups, a volcano plot analysis was used with the following parameters: FDR threshold 0.05, S0 0.1. All proteins above the curve on the left or right are significant.

## Results

### Targeted sequencing identifies an *IFT74* exon 2 deletion in patients with a combined PCD and primary ciliopathy phenotype

**Figure 1:**
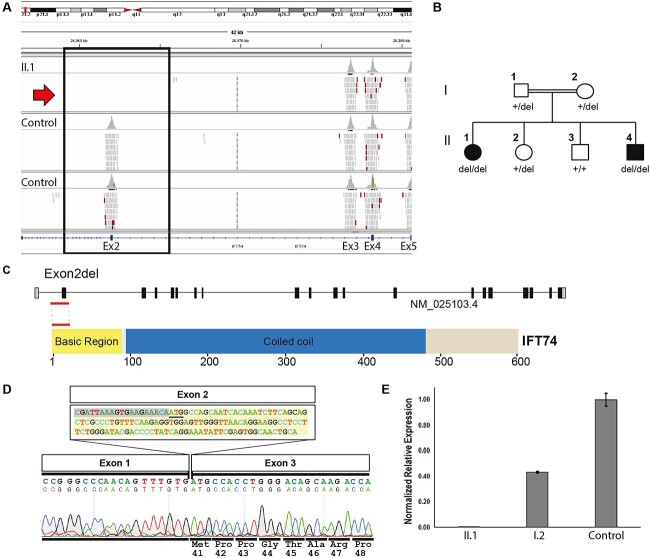
An intragenic *IFT74* exon 2 deletion in a syndromic PCD family predicted to remove the initiation codon and N-terminal 40 amino acids. (**A**) Integrated Genomics Viewer (IGV) screenshot of targeted sequencing generated BAM file of II.1, the affected girl in a Palestinian family with syndromic PCD, aligned to the reference genome (GRCh37/hg19) in comparison to two control samples in the same sequencing run. No reads were detected for exon 2 in II.1, indicating a homozygous deletion of this entire first coding exon of *IFT74*, with a uniform coverage of other *IFT74* exons detected among all samples. (**B**) Familial segregation analysis in the pedigree of II.1 detected the same homozygous exon 2 deletion in a younger affected sibling II.4, carrier status in both parents and an unaffected sibling II.2, with another unaffected sibling II.3 not carrying the deletion. Males and females shown by squares and circles respectively; filled symbols indicate the PCD affected individuals. (**C**) *IFT74* genomic organization (above) and the coded protein product (below), showing location of the exon 2 deletion affecting the start of the N-terminal basic region. (**D**) RT-PCR confirms that the exon 2-spanning genomic deletion only deletes exon 2 from the *IFT74* mRNA. Sanger sequencing of a nasal brushing cDNA sample from II.1, the *IFT74* exon 2 deletion patient, using primers in the 5’ UTR and exon 3 of *IFT74* shows a complete deletion of exon 2 the first coding exon, with preserved exon 3, resulting in an inframe deletion of the initiation codon and the first 40 N-terminal amino acids, predicting initiation of a shortened N-terminus-deleted IFT74 from methionine 41 at the start of the intact exon 3. In the deleted exon 2 sequence, the original native start codon is underlined and the deleted coding region is highlighted (**E**) Allele-specific RT-qPCR of the nasal brushing cDNA sample from II.1 using primers spanning *IFT74* exons 2 and 3 to detect exon 2-carrying *IFT74* transcripts, confirmed lack of expression in the affected child II.1 and expression around halved in the carrier mother (I.2) compared with healthy control. Error bars indicate SEM (standard error of the mean) based on each PCR reaction being done with technical triplicates.

As part of a project to investigate the genetic and clinical spectrum of PCD in Palestine, individuals admitted to paediatric pulmonology clinic with symptoms suggestive of PCD were subject to diagnostic testing by nasal nitric oxide (nNO) measurement, transmission electron microscopy (TEM), and genetic testing, as recently reported (36). In this analysis, targeted gene panel sequencing with an established bioinformatics pipeline, coupled to CNV analysis using ExomeDepth software (37), revealed a homozygous genomic deletion spanning the whole of exon 2 of *IFT74*, in one child (II.1) with suspected PCD and syndromic features from a Palestinian Arab family (Fig. 1A). Variant analysis did not prioritize any other DNA variants of interest based on rarity (MAF ≥ 0.01) or with additional focussed filtering of variants in a panel of >300 PCD and candidate cilia genes (Supplementary Material, Table S1). The parents in this family were consanguineous, and there was a younger child affected with similar features, in addition to two unaffected siblings (Fig. 1B). It was possible to obtain DNA samples from the entire family, and in follow-up Sanger sequencing screening, a recessive pattern of inheritance of the *IFT174* exon 2 deletion was detected that is consistent for most forms of PCD motile ciliopathy disease since it was verified that the second (younger) affected child (II.4) also carried the deletion in homozygous state, while both parents and an unaffected sister were heterozygous carriers and an unaffected brother did not carry the mutation (Fig. 1B).

This exon 2 deletion was not found in CNV analysis of 180 other unrelated Palestine PCD patient and control samples screened in the project, and it was also indicated to be rare in the wider population, as it is not found in gnomAD structural variants or among structural variants reported in the Database of Genomic Variants (41). However, a homozygous *IFT74* exon 2 deletion variant had recently been reported by Hammarsjö *et al*. to be the disease cause in a rare skeletal ciliopathy case with early lethality owing to thoracic hypoplasia and respiratory insufficiency (motile cilia disease was not studied in this case, owing to neonatal lethality) (42). This previous study detailed the genomic breakpoints of the exon 2 deletion with the variant reported as a 3 kb deletion, ClinVar variant ID 984954: NM_025103.4(IFT74): c.-19-2025_120 + 884delinsTTA. By designing PCR primers within intron 1 and intron 3 positioned outside these breakpoints at each side of this deletion, followed by Sanger sequencing, we could confirm that the exact same homozygous intragenic deletion breakpoints spanning 3046 base pairs [chr9: g.26959924–26 962 971 (GRCh38)] were the cause of exon 2 deletion in both the affected patients in our family; carrier status of the parents was also established, thereby confirming a recessive inheritance pattern (Fig. 1B, Supplementary Material, Fig. S1). The country of origin of the previous family in Hammarsjö *et al*. was not stated, but it was a consanguineous union, and this could be a recurrent recessive mutation that is ancestrally shared.

To determine the effect of the exon 2-spanning 3-kb genomic deletion on the expression of *IFT74* mRNA in these affected patients, nasal brushings were obtained from available family members in order to extract mRNA samples from their motile ciliated respiratory epithelium. RT-PCR in these samples followed by Sanger sequencing of the amplified band in the affected patients was performed. This revealed that the patient’s transcripts had a complete loss of exon 2 only, which is the first coding exon of *IFT74* containing the start codon; hence, this deletion is located at the N-terminus of the expressed IFT74 protein (Fig. 1C). There was no deletion effect on exon 1 (non-coding exon) or on exon 3, such that in the expressed *IFT74* cDNA transcripts amplified from patient cells, the end of exon 1 is directly joined to the start of exon 3 (Fig. 1D). Hence, the predicted result of the genomic exon 2 deletion is an inframe loss of the first 40 amino acids from the N-terminus of the encoded 600 amino acid IFT74 protein and an inframe N-terminal-deleted 560 amino acid protein initiated from methionine 41 located right at the start of exon 3 (Fig. 1D). Quantitative qRT-PCR using *IFT74* exon 2-specific primers confirmed the very low abundance (absence) of any transcripts that included exon 2 in the affected patient II.1, with approximately halved levels of expression of exon 2–deleted transcripts in the carrier mother I.2 compared with the normal expression level found in a healthy control (Fig. 1E).

**Figure 2:**
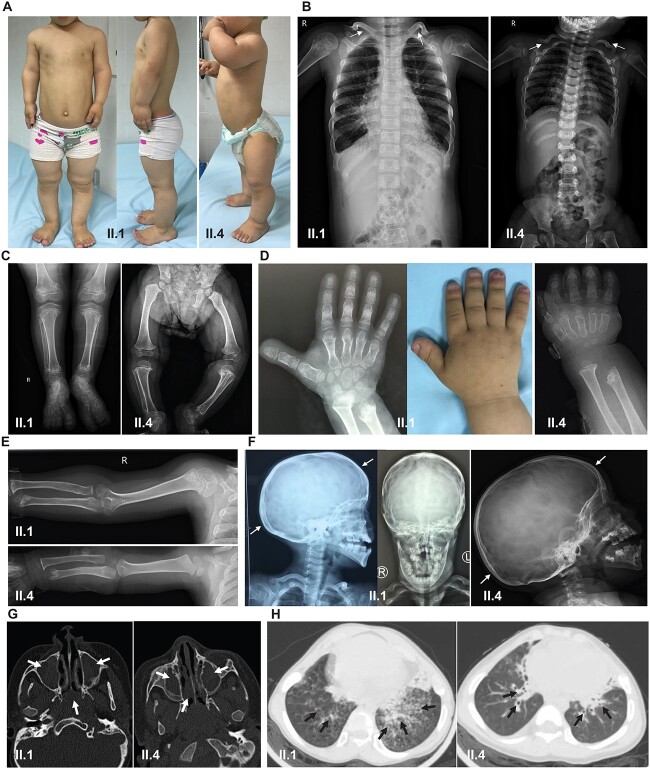
Clinical characteristics of individuals with biallelic *IFT74* exon 2 deletion mutations indicating both skeletal dysplasia and respiratory symptoms. (**A**) Both affected children II.1 (aged 8) and II.4 (aged 2) showed abnormal bone development with a short stature characterized by shortened arms and legs reminiscent of certain skeletal ciliopathies, bowing of the legs, which was more prominent in the older sibling II.1, and spine curvature (lordosis) more evident in II.4. Chest X-rays showed malformed clavicles (arrows) and narrowing of the ribs and thorax, the thorax in II.4 being slightly more narrowed, and bell shaped (**B**). X-rays show the shortened long bones with widened ends of the long bones (epiphyses) affecting both legs (**C**) and arms (**E**), and mildly shortened digits (brachydactyly) without polydactyly (extra digits) of the hands and feet (**A**, **D**) affecting both siblings. Cranial X-ray showed that dolichocephaly also affected the skull in both siblings, i.e. elongated skull diameter, front to back as indicated by arrows (**F**). (**G**) Both affected siblings II.1 and II.4 had symptoms of motile cilia disease shown in axial-view CT scans demonstrating swollen, inflamed sinuses and mucus blockage (arrows). (**H**) Chest X-ray of II.1 and II.4 shows features of bronchiectasis including enlarged airways and bronchi with mucous accumulations (arrows).

*IFT74* mutations were previously reported to cause a spectrum of primary ciliopathy phenotypes characterized by skeletal involvement diagnosed variously as Bardet–Biedl, Joubert, and Jeune syndrome (42–47). Both the affected children in this family have a complex phenotype that has features unique from this previously reported clinical spectrum. There are some overlapping features of severe but non-lethal skeletal dysplasia in addition to retinal dysplasia; however, they also both displayed clinical symptoms of PCD. The siblings shared similar features of short upper and lower limbs with bowing of the legs and exaggerated lordosis of the spine, resulting in a disproportionate short stature that is suggestive of the Jeune syndrome/short-rib thoracic dysplasia subtypes of ciliopathies (Fig. 2A). The elder girl’s height was at −5.8 SD (standard deviation) at the age of 8 years, and the younger boy’s height was at −6.8 SD at the age of 3 years, based upon WHO growth curves appropriate for each age and gender. In chest X-ray, both siblings showed abnormal skeletal findings, with narrowing of the thoracic cavity owing to shortened ribs, accompanied by ‘handlebar’ clavicles (Fig. 2B). X-rays revealed shortened long bones in the arms and legs with widened metaphyses (Fig. 2C and E), in addition to brachydactyly in the hands and feet (Fig. 2A and D). Both siblings also displayed dolichocephaly, which is more a feature of cranioectodermal dysplasia/Sensenbrenner syndrome (Fig. 2F). Neither sibling had any neurological symptoms to warrant further neurological testing. No polydactyly or renal problems were noted, but both patients had retinitis pigmentosa, which did not result in blindness but conferred impaired vision at night.

These features are in keeping with the skeletal dysplasia phenotype of the previously *IFT74* homozygous exon 2 deletion case reported by Hammarsjö *et al*.; however they did not report retinal dysplasia in their case. Furthermore, the affected siblings in our family were admitted to a pulmonary clinic with novel features for *IFT74* mutations of severe pulmonary disease. Both II.1 and II.4 had a history of neonatal respiratory distress, chronic productive cough, rhinosinusitis, otitis media, recurrent pneumonia, and recurrent bacterial infections, and both have had repeated hospital admissions in keeping with PCD. Mucus accumulation was evident in the swollen sinuses (Fig. 2G). No situs inversus or cardiac abnormality was noted in either of them. The older affected girl showed a very low nasal NO level dyskinesia of 4.5 nl/min compared with healthy control subjects, that is consistent for PCD, but this could not be measured in the affected boy owing to his young age. Both affected siblings also displayed signs of bronchiectasis with severe symptoms of lung disease in their lung computed tomography (CT) (Fig. 2H).

### *IFT74* exon 2 deletions result in ciliogenesis defects that impact upon motile cilia in ciliated respiratory epithelial cells of affected individuals

**Figure 3:**
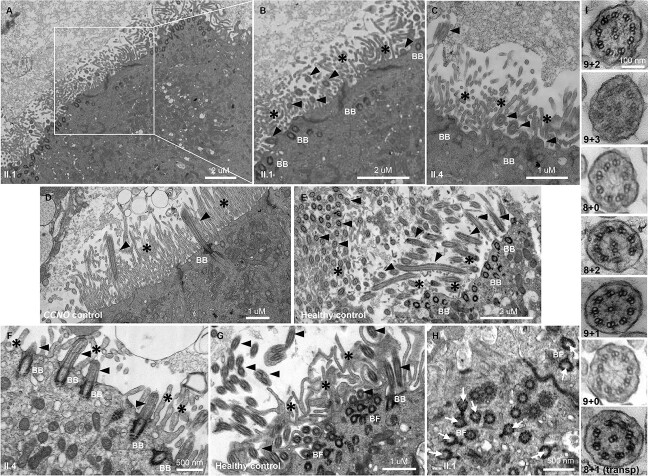
Individuals with biallelic *IFT74* exon 2 deletion mutations have shortened airway epithelial multicilia with internal microtubular defects. (**A**–**C**) Transmission electron microscopy of nasal epithelial cells from affected siblings II.1 (**A**, and white square magnified in **B**) and II.4 (**C**), showing the epithelial surface with respiratory motile cilia that are markedly short and reduced in number, the cilia axonemes visible with reduced frequency than usual, and extending to lengths terminating close to the apical cell surface (as marked by the presence of microvilli). In comparison, healthy individuals have plentiful cilia with axonemes extending much further from the epithelial surface, seen in side view and cross sections in (**E**). Note in (**D**, **E**) that the longitudinal sections of cilia and positioning of the basal bodies from healthy volunteers are representative of usual observations, and it can be seen that owing to plane of sectioning full axonemal length may not be visible (arrowheads showing the base and tips of cilia). In both siblings, the shortened cilia appeared to have normal looking basal body structure and alignment, as seen along the apical surface of the cells. The short cilia of the patients were clearly visible in side view (**F**) compared with control (**G**). (**D**) In contrast, nasal epithelial cells from a PCD patient carrying *CCNO* ciliogenesis mutations had very infrequent cilia but these were full length, with fields of microvilli seen in isolation at the ciliated cell apical surfaces. (**G**, **H**) Disorientation of the basal feet of the cilia in the *IFT74* mutation patients was also seen, compared with control (foot misorientation shown by white arrows in H). Cross sections taken through the cilia axonemes showed that in contrast to the 9 + 2 microtubular array of normal axonemes (**I**, top panel), a variable range of internal ciliary axoneme ultrastructural defects was present in the patients, dominated by microtubular defects including absence of one or both central pair microtubules (**I**, lower panels). BB; basal body, BF; basal foot; black arrowheads, cilia axonemes in longitudinal or cross section; asterisks, microvilli; white arrows, cilia foot direction; transp, transposition of peripheral microtubules to the centre.

Transmission electron microscopy (TEM) analysis was performed on three brushings (nasal and bronchial) from the individual II.1 and two brushings from II.4. This survey showed clearcut airway motile cilia defects that were consistent for both affected siblings, with shortened respiratory cilia present in reduced numbers on the apical surfaces of the airway ciliated epithelial cells (Fig. 3A–C and F), compared with the long and abundant cilia seen in multiciliated epithelial cells in the airway of healthy control individuals (Fig. 3E and G). Notably, this also differs to the airway ciliary defects in patients with PCD caused by ciliogenesis defects, e.g. those arising from *CCNO* mutations, where sparse cilia numbers are also found (48–50). PCD patients carrying *CCNO* mutations typically have cilia that are highly reduced in numbers but full length, not shortened, and basal bodies that can be mislocalized (Fig. 3D).

In cross sections of the shortened motile airway cilia of individuals II.1 and II.4, the typical 9 + 2 axoneme ultrastructure was disrupted with the ciliary microtubules often disarranged and a range of disrupted internal axonemal architectures recorded. The abnormalities mainly affected the microtubules that had abnormal numbers of central pairs and/or peripheral doublets (being fully absent from a cilia cross section or having reduced or increased tubules), microtubular translocations, and loss of microtubular integrity; hence 8 + 0, 8 + 1, 8 + 2, 9 + 0, 9 + 1 and 9 + 3 axonemal arrays were all observed, instead of the usual 9 + 2 (Fig. 3I). There was also some disrupted orientation of the cilia basal feet, which is normally determined by the integrity of the ciliary microtubular structure and the successful generation of cilia fluid flow (Fig. 3G and H) (51,52). The localization and ultrastructure of the basal bodies, where visible, appeared to be normal in longitudinal sections (Fig. 3B, C, F, and G). We note that owing to the reduced number of cilia in these patients and the short length of cilia, only a low number of cilia cross sections could be analysed for microtubule defects per patient; however, a majority that were analysed had a microtubule/axonemal defect.

**Figure 4:**
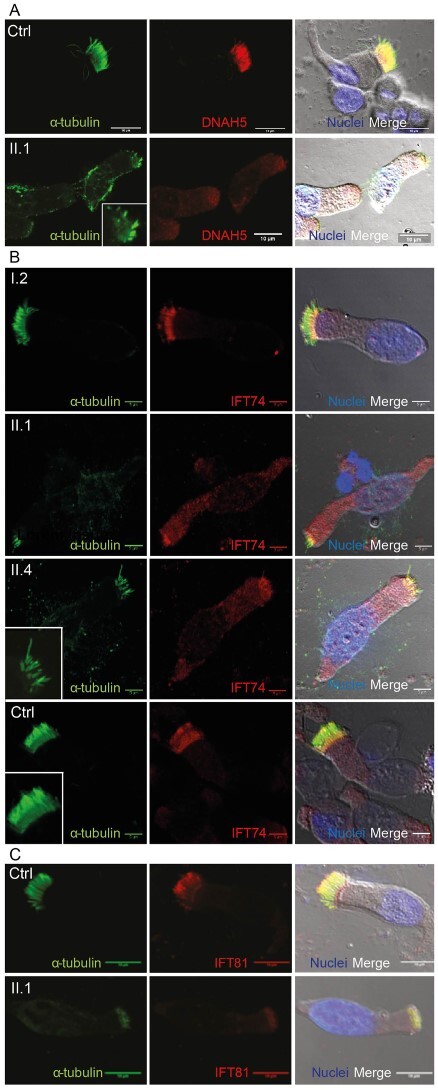
Airway epithelium of individuals with *IFT74* exon 2 deletions has reduced numbers of shortened airway motile cilia and disrupted cilia protein distributions in the multiciliated cells. (**A**) In individual II.1 homozygous for exon 2 *IFT74* deletions (lower panel and magnified in inset), immunofluorescence staining of the outer dynein arm protein DNAH5 (red) in respiratory cilia from a primary nasal brushing sample showed greatly reduced overall levels of DNAH5, found in cilia where few looked close to normal length and most were short; there was also a reduced number of cilia stained using the axonemal microtubular marker acetylated α-tubulin (green). In comparison DNAH5 had a normal distribution along the entire length of the multicilia in controls (upper panel). In the patient’s cells, DNAH5 is seen to accumulate in the cell body notably at the apical cytoplasm underneath the cilia. Scale bars, 10 um. Full blot shown in Supplementary Material, Fig. S3. (**B**) In a healthy control (bottom panel) and the family’s carrier mother (I.2, top panel), immunofluorescence of ciliated cells in a primary nasal brushing sample showed in both, that IFT74 (red) has a similar distribution to DNAH5, being evenly distributed along the length of the multicilia axonemes co-localizing with the cilia marker acetylated α-tubulin (green). In the affected individuals II.1 and II.4 homozygous for exon 2 *IFT74* deletions (middle panels), IFT74 immunostaining had an abnormal distribution with reduced levels of protein and with more IFT74 apparent within the cytoplasm and especially accumulated in the apical cytoplasm beneath the cilia. Immunofluorescent staining of IFT74 was still evident along the short, infrequent patients’ cilia, compared with its normal distribution along the cilia of the mother and healthy control individual (inset panels in II.4 and control). Scale bars, 5 um. (**C**) In comparison, immunofluorescence staining of IFT81, the IFT-B binding partner protein of IFT74, was also very reduced in the patients’ cells but still co-localized with the cilia marker acetylated α-tubulin (green) along the short, sparse patients’ cilia, compared with its normal distribution along the cilia of a healthy control. Scale bars, 10 um.

Immunofluorescent staining of motile cilia obtained from the nasal brushing biopsies of the patients confirmed the TEM findings with markedly sparse, shorter cilia in the patients’ cells compared with healthy controls. There were only occasional more normal-looking cilia present as a visible extension at the apical surface of the ciliated cells. This was clear in staining for the outer dynein arm marker protein DNAH5 (ODA component), which highlighted mostly short cilia and showed reduced levels of DNAH5 immunofluorescence in the patient’s cells compared with its normal distribution levels along the whole length of the ciliary axonemes of the healthy control, overlapping with the cilia microtubular marker–acetylated alpha tubulin (Fig. 4A). Reduced DNAH5 levels could solely be reflecting the shortened cilia of the patients, but it also appeared somewhat reduced in intensity within the short cilia. There was also a suggestion of aberrantly accumulated DNAH5 protein in the cytoplasm of the IFT74 patient’s cells compared with being absent in the cytoplasm of cells from the carrier mother or controls, suggesting possibly delayed ciliary import (Fig. 4A). Staining for IFT74 highlighted the patient’s short and sparse cilia, with abnormal distribution in the patients’ cells as IFT74 was more highly present in the cytoplasm, and within the cell body, it was visibly accumulated at the apical cytoplasm, like DNAH5 (Fig. 4B). IFT74 is present along the axonemes of motile airway cilia in controls, and in comparison, IFT74 did appear to co-stain the remnant cilia in the patient’s cells, co-localizing with acetylated alpha tubulin. It is possible to speculate that mutant *IFT74* transcripts lacking exon 2 still express levels of a protein that resists degradation and is capable of ciliary import (Fig. 4B). To look at the effect of IFT74 mutations, we immunostained for another IFT-B complex component, IFT81, which heterodimerizes with IFT74 to jointly form an IFT-B tubulin-binding module (53). In the patients’ cilia, IFT81 showed a similar localization in the ciliated cells to controls suggesting evidence of retained cilia import, but with a marked reduction in its axonemal staining (Fig. 4C). Additional staining for markers of IFT-B, IFT-A and the retrograde IFT motor showed a reduction in their cilia localization, with the IFT-B protein IFT88 notably reduced; this also showed that retrograde IFT-A components may be disrupted because of IFT74 deficiency and disrupted anterograde IFT-B transport (Supplementary Material, Fig. S2). However, with the shortened, stubby cilia, definitive interpretation is not possible without a more quantifiable approach, and samples were not available for patient respiratory epithelial cultures to study IFT and ciliogenesis more closely.

### *IFT74* exon 2 deletions result in expression of truncated IFT74 proteins and perturbed IFT-B gene transcription in ciliated respiratory epithelial cells of affected individuals

**Figure 5:**
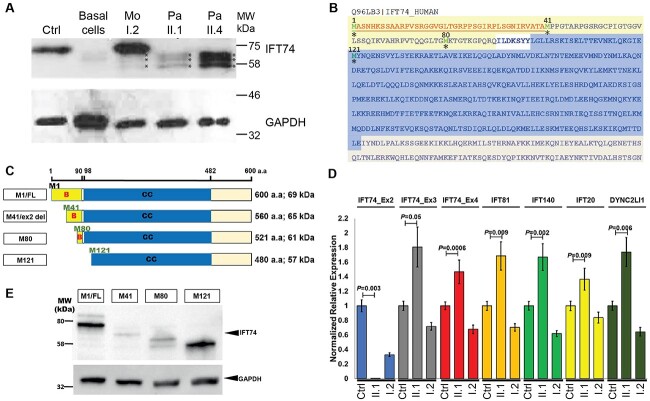
*IFT74* exon 2 deletions result in expression of truncated protein and altered transcription of IFT-B complex genes. (**A**) Western blotting of protein lysates extracted from nasal brushing biopsies of individuals carrying homozygous *IFT74* exon 2 deletions using a published anti-IFT74 antibody (27334–1-AP, Proteintech), revealed lower-molecular-weight protein products present in the affected individuals II.1 and II.4, but not in a healthy control (Ctrl), which showed a full-length IFT74 protein band at ~69 kDa. This band was also present in the carrier mother, I.2, but absent from basal epithelial cells cultured from a healthy control representing pre-ciliogenesis (non-ciliated) airway precursor cells (54). The affected siblings lacking full-length IFT74 have three smaller-molecular-weight bands hypothesized to be N-terminus truncated protein products arising from protein translation initiation events downstream from the exon 2-deleted start methionine of IFT74, running at ~65, 61, and 57 kDa. The three bands are also present at reduced levels in the carrier mother (asterisks). Anti-GAPDH was used as a loading control on the same samples. (**B**) The IFT74 canonical protein isoform is comprised of 600 amino acids (UniProtKB Q96LB3). Exon 2 codes for the first N-terminal 40 amino acids (deleted amino acid sequence coloured red in bold underlined). Possible inframe downstream methionine residues used as alternative translation starts in IFT74 exon 2 deletion patients are highlighted. (**C**) IFT74 protein domains showing structure of the Strep-FLAG-tagged full-length and deletion expression constructs used in mass spectrometry (similar colours have been used to highlight the IFT74 proteins domains in B and C). Full-length IFT74 protein has a basic region (1–90 a.a) and a coiled coil domain (98–482 a.a) with a molecular weight of 69 kDa. After exon 2 deletion, if translation starts at methionine residue at positions 41 or 80, this would only affect the basic region, but if translation started at methionine residue 121 or more downstream positions, this would also disrupt a part of the coiled coil domain. (**D**) Transcriptional upregulation of *IFT74* downstream to the deletion and other IFT components. RT-qPCR was used to assess the expression levels of *IFT74* transcripts and other IFT components (*IFT81, IFT20, IFT140*, and *DYNC2LI1*) normalized to *GAPDH* quantification, in nasal brushing biopsies obtained from the patient, mother, and a healthy control. There were elevated expression levels of all genes quantified in the patient’s sample (II.1), but no amplification when using primers in *IFT74* exon 2, while the carrier mother (I.2) had expression levels similar to controls (Ctrl). (**E**) Western blotting analysis of the IFT74 expression constructs shown in (**C**) transfected into HEK293 cells, after affinity purification using anti-FLAG. Protein of the correct molecular weight was pulled down for all constructs, but the quantities of IFT74 pulled down for two of the constructs, exon 2 deletion and M80 (∆79 a.a), were lower than that pulled down for full length and M121 (∆120 a.a) constructs.

Using primary multiciliated respiratory epithelial cells collected from patients via nasal brushing samples, we proceeded to investigate the consequences on motile cilia function of the *IFT74* exon 2 deletion. Western blotting was performed on protein lysates extracted from nasal brushings from the affected siblings II.1 and II.4, individual I.2 (heterozygote carrier mother), and a healthy control from the same ethnicity and geographical region. This showed a band corresponding to the expected size of full-length IFT74 protein (69 kDa) in the mother and healthy control samples, which was absent from the homozygous exon 2 deletion patients’ samples. Instead, II.1 and II.4 both had three smaller bands running at lower- molecular-weight sizes than full-length IFT74 (Fig. 5A). These three smaller mutant protein products were also faintly noted in the sample from the mother who carries one copy of the IFT74 exon 2 deletion variant.

IFT74 (UniProt Q96LB3) has two protein isoforms produced by alternative splicing: isoform 1, the canonical isoform (Q96LB3–1) formed of 600 amino acids with a molecular weight of 69 kDa and isoform 2 (Q96LB3–2), which has 372 amino acid length and molecular weight 42 kDa. Both protein isoforms are identical in the first 351 amino acids including the first 40 amino acids corresponding to exon 2. These smaller proteins identified in the patients’ cells potentially represent three inframe truncated IFT74 isoform 1 protein products, wherein it is hypothesized that the biggest truncated patient protein could correspond a shortened isoform expressed from methionine 41 (M41) at the start of exon 3 (Figs 1D and 5B), which would therefore consist of 560 instead of the full-length 600 amino acids, lacking the first 40 N-terminal amino acids. As there are methionine residues further downstream, the next closest being located at positions M80 and M121 (Fig. 5B), we could further propose that these explain the presence of additional smaller truncated protein products in the patients’ samples. *In silico* prediction using each of these three downstream methionines as a translation start codon downstream to the deletion, would yield protein products of estimated molecular weights 65, 61, and 57 kDa (Fig. 5C). IFT74 protein has a basic region extending between amino acids 1 and 90 and an extended coiled coil domain that extends between amino acids 98 and 482. Exon 2 deletion (∆40 amino acids) is expected to affect the N-terminal basic domain only. The translation machinery might skip the first AUG and use these further downstream AUGs coding methionine residues, most likely M80 (∆79 amino acids and only affecting the basic region) or M121 (∆120 amino acids, which would delete the basic domain and disrupt a part of the coiled coil domain), or potentially at even more downstream positions (Fig. 5C).

The transcriptional consequences of the *IFT74* exon 2 deletion were also assessed, by qRT-PCR on mRNA extracted from the nasal brushings of the affected sibling II.1, their mother (I.2) and a healthy control from the same ethnicity and geographical region, using specific forward primers designed in exons 2, 3, and 4 amplified with a reverse primer designed to span a downstream exon–exon junction. The forward primer in exon 2 re-confirmed the low abundance of exon 2-containing *IFT74* transcripts in the II.1 patient’s sample, the I.2 mother’s sample (heterozygous carrier) expressing about half levels of exon-2-containing *IFT74* transcripts compared with healthy controls. Using primers in exons 3 and 4, *IFT74* transcripts downstream to the deletion were then amplified. These were present at similar proportions in the control and carrier mother, while in the patient, they were also present, thereby indicating that in the patient’s cells, exon 2-deleted transcripts are present. The patient, in fact, had higher expression levels (nearly doubled), compared with the mother and healthy control, suggesting the potential of a possible feedback compensatory mechanism where *IFT74* transcriptional upregulation is used by the cells to try to overcome the potential defective function of IFT74 secondary to the deletion (Fig. 5D). Next, the relative mRNA expression levels of other IFT genes were also quantified. In qRT-PCR of other IFT-B genes (*IFT81* and *IFT20*), an IFT-A gene (*IFT140*) and *DYNC2LI1*, a gene encoding a component of the retrograde IFT motor, these were all expressed with a similar pattern to that of the *IFT74* gene downstream of exon 2, i.e. higher levels in the patient compared with the control, with expression lower in the carrier mother than the control as she carries only one copy of the full-length *IFT74* (Fig. 5D). This indicates a perturbation of wider IFT transcript expression patterns in *IFT74* exon 2 deletion patient cells; however, the underlying nature of that perturbation is not yet clear.

### Protein complex analysis of IFT74 exon 2 deletion mutants shows loss of an essential role of the first 40 basic domain amino acids in stability of IFT-B complexes

To better understand the mutational consequences of the exon 2 deletion and the existence in exon 2 deletion patients’ cells of retained truncated forms of the protein, we generated Strep-FLAG-tagged constructs corresponding to full-length IFT74 (UniProt Q96LB3) expressed from the native methionine M1 and a truncated IFT74 initiated from M41 to mimic the exon 2 deletion mutation. In addition, constructs were made with IFT74 initiated at the next two downstream methionines M80 and M121, according to the hypothesis that these smaller N-terminal-deleted forms might also be expressed in exon 2 patient cells (Fig. 5C). The effect of exon 2 and these larger N-terminal deletions on IFT74 interactions was then studied using affinity purification coupled with mass spectrometric LC–MS/MS analysis. HEK293 cells were transfected with the different Strep-FLAG-tagged IFT74 constructs or a negative control (RAF1 Strep-FLAG-plasmid) in six replicate experiments each. RAF1 was analysed rather than the empty vector, as it is a validated non-ciliary control protein of similar size (648 amino acids) and recombinant expression levels to IFT74, with known interactors previously detected by mass spectrometry (55). Transfection was followed by anti-FLAG immunoprecipitation (IP) of the fusion proteins to pull down their binding partner proteins, with subsequent mass spectrometry and label-free quantification (LFQ) of the IP elute proteomic data. First, all the constructs were confirmed to express detectable levels of IFT74 protein in HEK293 cells sufficient to proceed with LC–MS/MS proteomics, by western blotting of transfected cell lysates. The blots used an IFT74 rabbit polyclonal (27334–1-AP, Proteintech) raised against a peptide of amino acids 151–350 within the CC domain; hence, this will bind to all the mutant proteins (Fig. 5E). Expression of the M41 (exon 2 deletion) and M80 mutant constructs was found to be reduced compared with full-length IFT74, with the M121 mutant construct found to be more stable and at equivalent expression levels to full-length IFT74.

**Figure 6:**
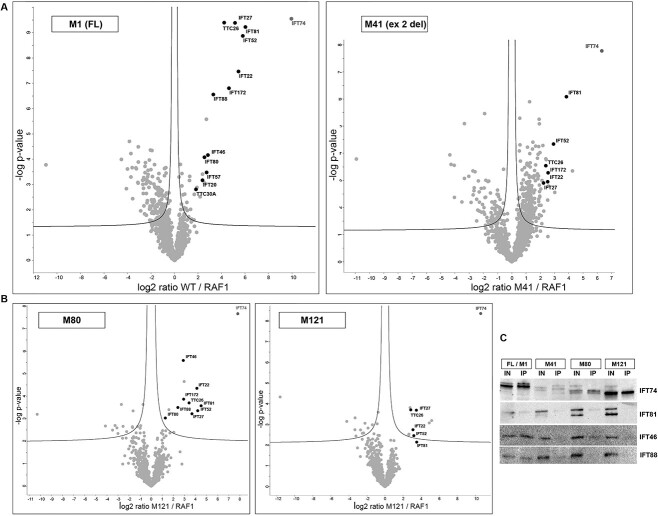
The IFT74 N-terminus is essential for stable binding of the ciliary IFT-B complex. (**A**) Volcano plot of the IFT74-interacting proteins enriched in affinity purification of IFT74 interactors after either full-length or exon 2 deleted mutant IFT74 (M41 initiation codon) was compared with RAF1 vector transfection of HEK293 cells. Protein components of the IFT-B complex that are enriched with the full-length or M41 mutant IFT74 are highlighted in black with name labels. Statistical significance was determined by permutation-based false discovery rate (FDR)-corrected *t*-test, threshold: *P* = .05 and S0 = 0. (**B**) Volcano plot as per (**A**), of the IFT74-interacting proteins enriched in affinity purification of IFT74 interactors after mutant IFT74 initiated at M80 or M121 was compared with RAF1 vector transfection of HEK293 cells. (**C**) Western blotting analysis of IFT74 affinity purification experiments using anti-FLAG, showing quantities of IFT74 interactors pulled down after transfection of the full-length versus the exon 2 deletion (M41), M80, and M121 mutant constructs. IFT81, IFT88, and IFT46, three representative IFT-B components, were fully or largely absent not being significantly pulled down by any of the N-terminal truncated proteins, compared with full length. Transfections and pull downs followed by western blotting were done six times with consistent results in all experiments.

To study the consequences of IFT74 N-terminus truncations, a volcano plot analysis was performed between different groups of experiments. Using (log2) of LFQ values generated a Gaussian distribution of the data, allowing imputing of missing values by normal distribution (width = 0.3 and down shift = 1.8) assuming that these missing data are close to the limit of detection (56). The false discovery rate (FDR) cut-off was set at 0.05 (a test result below 0.05 is considered significant) and S0 (artificial within groups variance) set at 0, as we performed six replicates for each construct with the main determinant of the significance statistics based on *P*-values (57). A comparison of LFQ values of the pulled-down proteins for full-length IFT74 versus the RAF1 control identified a large set of 168 significantly and robustly enriched proteins, representing potential IFT74 interactors of interest for further comparative analysis between the full-length and various mutant constructs (Supplementary Material, Table S4). A set of 13 IFT components of the IFT-B complex were enriched in the samples where full length of IFT74 was transfected, compared with the empty vector transfection: IFT74, IFT172, IFT81, IFT22, IFT52, IFT27, IFT56, IFT46, IFT88, IFT57, IFT20, IFT80, and IFT70A (Fig. 6A, Table 1).

**Table 1:** Summary of IFT74 protein interactors enriched by anti-FLAG IP. RAF1, control RAF1 vector; FL, full-length IFT74; M41, M80, and M121 indicate N-terminal truncation mutant constructs. Grey boxes with n.d. indicate not defined, i.e. proteins that were not enriched at levels detectable in enough replicates in the experiments.

Protein IDs	Name	WT/RAF1 log2 ratio	M41/RAF1 log2 ratio	M80/RAF1 log2 ratio	M121/RAF1 log2 ratio	M121/WT log2 ratio	WT/RAF1 log *P*-value	M41/RAF1-log *P*-value	M80/RAF1-log *P*-value	M121/RAF1-log *P*-value	M121/WT-log *P*-value
Q96LB3	IFT74	9.95	6.36	7.81	10.59	0.63	9.55	7.80	7.67	8.33	.52
Q9UG01	IFT172	4.63	2.55	2.93	n.d.	-3.38	6.81	3.28	3.87	n.d.	6.34
Q8IY31	IFT20	2.39	n.d.	n.d.	n.d.	-1.89	3.17	n.d.	n.d.	n.d.	2.02
Q9H7X7	IFT22	5.45	2.51	4.11	3.08	-2.83	7.47	2.95	4.35	2.75	2.51
Q9BW83	IFT27	5.16	2.23	3.67	3.47	-1.90	9.39	2.91	3.22	3.70	1.35
Q9NQC8	IFT46	2.84	n.d.	2.89	n.d.	-1.24	4.16	n.d.	5.59	n.d.	2.11
Q9Y366	IFT52	5.82	2.92	4.20	3.19	-2.53	8.87	4.34	3.35	2.46	2.03
A0AVF1	IFT56/TTC26	4.22	2.38	3.42	2.84	-1.72	9.39	3.54	3.70	3.71	2.69
Q9NWB7	IFT57	2.73	n.d.	n.d.	n.d.	-0.94	3.48	n.d.	n.d.	n.d.	0.62
Q9P2H3	IFT80	2.55	n.d.	1.28	n.d.	-0.68	4.07	n.d.	3.03	n.d.	0.71
Q8WYA0	IFT81	6.04	3.84	4.50	3.51	-2.21	9.23	6.08	3.57	2.15	1.31
Q13099	IFT88	3.30	n.d.	2.40	n.d.	-2.17	6.55	n.d.	3.50	n.d.	5.69
Q86WT1	TTC30A/IFT70A	1.83	n.d.	n.d.	n.d.	-0.43	2.81	n.d.	n.d.	n.d.	.38

Further analysis of LFQ values was then performed to detect the levels of these enriched IFT-B protein components in cells transfected with the three IFT74 mutant constructs, compared with the full-length IFT74 construct (Fig. 6A and B, Table 1). Many of the same IFT proteins were found to be reduced in the mutant samples, indicative of disturbed IFT-B interactions. The full length IFT74 as well as the M41 and M80 mutants were all able to bind the IFT-B complex, although the full IFT-B complex was only immunoprecipitated with full length (Fig. 6A and B, Table 1). Nevertheless, most of the IFT-B proteins were clearly also pulled down as interactors for M41 and M80 mutant proteins, indicating that these mutants are in principle still able to bind the IFT-B complex. However, these experiments identified much lower overall levels of interactors in the M41 (exon 2 deletion) and M80 mutant transfected cells, compared with full-length IFT74 and M121 amino acid constructs, likely owing to their lower expressed levels of IFT74 (Fig. 5E), but with evidence of slightly more disruption of IFT-B complexes associated with the exon 2 deletion mutant than M80 amino acid mutant construct (Fig. 6A and B, Table 1). IFT74’s partner protein IFT81 could still be pulled down by all three mutants, but at greatly reduced level, and as an example of other IFT-B proteins, IFT88 was either not detected as pulled down by the M41 and M121 mutants or was very reduced (M80 mutant), which fits with its absence from the cilia of the exon 2-deletion patients (Supplementary Material, Fig. S2).

LFQ values compared between the full-length protein and M121 mutant (disturbing a part of the coiled coil domain) showed something different for M121 because the IFT74 intensities were stable across all samples of both constructs but the M121 mutant expression resulted in more disturbance of the IFT74 interactions; this included more disrupted interaction with other IFT-B complex proteins than for either the exon 2 deletion or the M80 mutants (Fig. 6B, Table 1). For M121, only few IFT-B proteins were pulled down and to just above the limit of significance, despite M121 being expressed at similar levels to full-length protein, meaning that most IFT-B proteins were not co-precipitated with M121 at all.

Disrupted interactions with the IFT-B complex were then confirmed for all the IFT74 mutant proteins by western blotting (Fig. 6C). This showed that in contrast to full-length IFT74, none of IFT74 mutants were able to fully co-immunoprecipitate three representative IFT-B complex proteins (IFT81, IFT88, and IFT46). These IFT-B partner proteins were severely reduced or fully absent in pull downs using the IFT74 exon 2 deletion, M80 and M121 constructs, with a trend to being more fully absent in the M121 pull downs (Fig. 6C).

## Discussion

We describe a complex syndrome with combined features of both motile and primary ciliopathy that arises from a ~3 kb genomic deletion in the intraflagellar transport complex B gene *IFT74.* This deletion results in complete loss of exon 2 that contains the initiation codon, creating an inframe loss of the first 40 amino acids of the IFT74 protein. Two affected siblings, both homozygous carriers of this mutation, were admitted to a pulmonary clinic. They displayed severe pulmonary disease with features of the motile ciliopathy primary ciliary dyskinesia, marked by low nasal nitric oxide, neonatal respiratory distress, chronic respiratory infections, glue ear, rhinitis, sinusitis, and bronchiectasis. They also had significant skeletal dysplasia, which is not a typical feature of PCD but suggestive of a primary ciliopathy. Their syndromic features included short ribs and thoracic compression, disproportionate stature with shortened long bones, and skull elongation, in addition to retinal dystrophy.

Human *IFT74* mutations are rare but have been reported in recent years, as summarized in Supplementary Material, Table S5. The spread of variants across the gene, different mutation types involved, and lack of functional analysis except in a few cases (46) does not yet allow for very conclusive genotype–phenotype correlation. These previous *IFT74* mutations were described to cause different primary ciliopathy subtypes including both Bardet–Biedl syndrome (BBS) expressed with postaxial polydactyly, retinal degeneration, obesity, intellectual disability, and hypogonadism (43,44,47,58) and Joubert syndrome (JS) expressed with postaxial polydactyly, developmental delay, subtle cleft upper lip, and molar tooth sign of the brain but no retinal anomalies or significant obesity (46,59). These *IFT74* cases are not early lethal cases; they show no renal involvement and do not show the skeletal thoracic, short stature, or dolichocephaly features we report. Hence, these previously reported *IFT74* mutations may present distinct features from the two siblings with only a few potential clinical overlaps such as retinal dystrophy.

The siblings here instead resemble a previously reported single ciliopathy case carrying an identical homozygous *IFT74* exon 2 genomic deletion, reported by Hammarsjö *et al*. to display thoracic hypoplasia (Jeune syndrome–like) features (42). This child shared the narrowed thorax, short ribs, handlebar clavicles, dolichocephaly, brachydactyly, and short tubular bones, but, unlike our siblings, had died at 1 week of age. Notably, none of these three exon 2 deletion cases have polydactyly, which is common in Jeune syndrome. Their dolichocephaly also appears more reminiscent of another primary ciliopathy, cranioectodermal dysplasia. Together, these findings show that *IFT74* exon 2 deletion mutations can specifically give rise to Jeune syndrome–like skeletal dysplasia with variable lethality. Furthermore, our cases show that IFT74 disease caused by exon 2 deletion can cause both non-motile and motile ciliopathy features. Had there been longer survival, the previously reported child may have shown similar chronic respiratory disease features (42).

The nature of this complex ciliopathy is consistent with the role of IFT74 as a component of the IFT-B adaptor complex required for ciliary trafficking in both non-motile and motile cilia. IFT74, together with its partner protein IFT81, is essential for ciliogenesis, as they form a tubulin-binding module of IFT-B that specifically mediates the anterograde transport of tubulin within cilia (53,60,61). *IFT74* mutations have previously been reported to cause short and malformed sperm flagella, both in mouse mutants and associated with human male spermiogenesis defects (45,62). However, in patients with *IFT74* mutations, the airway motile cilia have never been examined. Here, we find short cilia of reduced number in the respiratory epithelium of our exon 2–deleted *IFT74* patients, which is revealed by ultrastructural TEM studies to have a hallmark of reduced, disrupted, and disorganized microtubules within the remnant cilia stubs. This appears to be consistent for a tubulin deficiency that we find to notably affect the central pair microtubules of the motile cilia.

Symptoms of motile cilia defects have also been reported before in selected primary ciliopathy syndromes. *OFD1* mutations known to cause oral–facial–digital syndrome type 1 can occur in patients with ciliary dyskinesia and situs inversus (32,34). Primary ciliary dyskinesia has been associated with X-linked retinitis pigmentosa in patients carrying *RPGR* mutations (33,63,64). Situs inversus defects can occur in humans with *IFT81* mutations and are found in mouse IFT-A mutants (65,66). Mutations in other IFT-B gene additional to *IFT74* (*IFT81*, *IFT20, IFT27, IFT172*), and in IFT-A genes (*IFT140*) can cause spermatogenesis defects and male infertility in mice (62,67–71). However, skeletal ciliopathy defects linked to human syndromic PCD forms have rarely been reported. In mice, *Spag17-* deficient mice lacking ciliary central pair microtubules and thus resembling a PCD cilia defect were found to have immotile airway cilia and PCD-like respiratory disease as well as skeletal abnormalities (72,73). One patient with a primary ciliopathy phenotype of cranioectodermal dysplasia arising from mutations in the IFT-A gene *WDR35/IFT121* was also reported to have motile ciliopathy symptoms of neonatal respiratory distress, recurrent respiratory infections, sparse and dyskinetic motile cilia, and situs inversus (74). The lack of detection likely reflects the rarity of these associations, but in skeletal primary ciliopathy patients such as short rib polydactylies and cranioectodermal dysplasia, their characteristically constricted thoracic cavity and compression of the lung and airways could also be a confounding association potentially masking motile ciliopathy signs (and causing early lethality).

Here, we were able to explore at the cellular level why exon 2 deletion of *IFT74* may cause a specific disease phenotype distinct from those conferred by other *IFT74* mutations. RT-PCR confirmed deletion of exon 2, splicing exon 1 and exon 3 directly together in transcripts expressed in primary respiratory epithelial cells from affected individuals. This deletes the normal start codon such that inframe protein translation could initiate from AUG^41^ directly at the start of exon 3, resulting in an expressed, shorter protein that lacks the first 40 N-terminal amino acids of the IFT74 basic domain. Consistent with this, western blotting of protein lysates from primary respiratory epithelial cells of affected individuals showed loss of the IFT74 full-length protein and expression of smaller products. The largest of these was consistent for molecular weight of a protein expressed from the inframe methionine 41 (M41), and these were hypothesized to potentially represent in-frame truncated IFT74 protein products produced from alternate translation start codons downstream to the deletion and secondary to a possible translation leaky scanning mechanism (75). These shorter forms could retain some IFT-B function, thus creating a hypomorphic mutation effect.

The IFT74 N-terminus is defined as the main protein domain required for intraflagellar transport of tubulin, as it directly binds to tubulin and tubulin transport in cilia is greatly reduced if this domain is removed (53,61). In *Chlamydomonas* mutant strains carrying an IFT74 N-terminus deletion, the IFT complex was still able to maintain some tubulin transport function compensated through the presence of normal levels of IFT81, the IFT74-partner protein (60,61). The reasons for more prominent skeletal involvement with *IFT74* exon 2 deletion compared with other reported *IFT74* mutations is not clear, but exon 2 deletion may represent a hypomorphic allele with residual function and, consequently, the clinical picture of the patients lies towards the milder end of the ciliopathy phenotypic spectrum. We also discovered through quantitative real-time PCR that in *IFT74* exon 2 deletion respiratory ciliated cells, there is a transcriptional upregulation of *IFT74* downstream to the deletion indicating dysregulation of the gene. Moreover, we found that other IFT gene transcripts are upregulated compared with healthy controls. This may reflect a possible compensatory feedback mechanism to overcome the defective function by certain ciliary genes in the *IFT74* network, a mechanism that has been reported before where a gene paralogue or a network of genes is upregulated in response to defective function of one gene, resulting in a milder disease phenotype (76,77).

We tested the hypothesis that smaller, inframe IFT74 proteins could be expressed in exon 2 deletion patients using the first and second downstream methionine residues (M41 and M80) that would affect the basic region of the IFT74 N-terminus, previously shown to be dispensable for the stability or the formation of the IFT-B complex (60,61). It was also previously shown that the IFT74 N-terminus interacts with the acidic tail of β-tubulin to increase the affinity of binding of IFT81 N-terminus to the globular domain of tubulin, for the purpose of IFT-mediated tubulin transport along the ciliary axoneme (53). The use of the next downstream AUG^121^ (M121) or any further downstream AUGs would affect not just the basic but also the coiled coil domain, through which IFT74 interacts with other IFT-B core proteins, e.g. interactions with IFT81 occur through the coiled coil domains of both proteins (53,78,79).

To determine the consequences of exon 2 deletion on IFT74 protein interactions with the rest of the IFT-B complex, affinity purification assay coupled with mass spectrometry and LFQ analysis was applied in cells transfected with full-length IFT74 and M41, M80, and M121 truncated mutants. For M41 and M80, most of the IFT-B proteins were clearly also pulled down as interactors, indicating that these mutants have potential to bind the IFT-B complex and therefore be functional. However, we suggest in light of the strong reduction in expression of these two mutants we saw, that it is likely that the abundance of fully assembled IFT-B complexes would be reduced and this would then lead to a hypomorphic cellular phenotype. The M121 mutant showed more disturbance of IFT74 interactions with other IFT-B complex proteins than for either the exon 2 deletion or the M80 mutants, even though M121 expressed at similar levels to full-length protein, indicating a more severe mutation effect. This is expected as it affects the coiled coil domain through which IFT74 interacts with its partner (53,78,79).

The two most common protein modifications that can affect protein homeostasis and possible degradation are N-terminal methionine cleavage and N-terminal acetylation. These two processes can occur either co- or post-translationally (80). This phenomenon is known as the N-end rule where the protein half-life depends on its N-terminal residue sequence and the modifications of its N-terminus (81–84). The presence of proline or threonine at position P2’ (the third amino acid position “M1, P1’, P2’…”) will hinder the N-terminal methionine cleavage resulting in protein degradation (85–87). This gives a possible explanation for the relatively greater instability of the IFT74 exon 2 deletion and M80 mutant seen in our over-expression experiments. M41 is followed by prolines in both the second and third residues, potentially preventing methionine cleavage and rendering this peptide readily available for degradation (Figs 1D and 5B). There is a threonine at position 3 (P2’) in relation M80 that similarly could lead to inefficient cleavage of methionine and degradation of this product (Fig. 5B). This is not the case if translation would move further downstream to start with the methionine at position 121; hence, for the M121 truncation mutant, the lack of proline or threonine at position P2’ could provide this smallest truncated protein with better stability.

In conclusion, this study identifies *IFT74* as a PCD-related gene, also characterizing affected individuals with an exon 2 deletion as a subtype of Jeune syndrome–like skeletal dysplasia. This expands the disease spectrum linked to *IFT74* mutations beyond primary ciliopathies, to include motile ciliopathy and respiratory disease, and this should be reflected in genetic screens of PCD patients in future. This analysis has helped to increase our understanding of the clinical and biological basis of respiratory ciliopathies, which may, in future, include additional cases of PCD and motile cilia disorders caused by mutations in *IFT74* and other IFT genes.

## Supplementary Material

Figure_1_revised_ddad132

Figure_2_revised_ddad132

Figure_3_revised_ddad132

Figure_4_ddad132

Figure_5_revised_ddad132

Figure_6_ddad132

Supplemental_data_2nd_revision_cleaned_ddad132

Supplementary_Table_S2_ddad132
