# Supplementary figures and images for: Defective airway intraflagellar transport underlies a combined motile and primary ciliopathy syndrome caused by IFT74 mutations
Source: Hum Mol Genet. 2023 Aug 9;32(21):3090–104. doi: 10.1093/hmg/ddad132 (PMC10586200; doi:10.1093/hmg/ddad132)

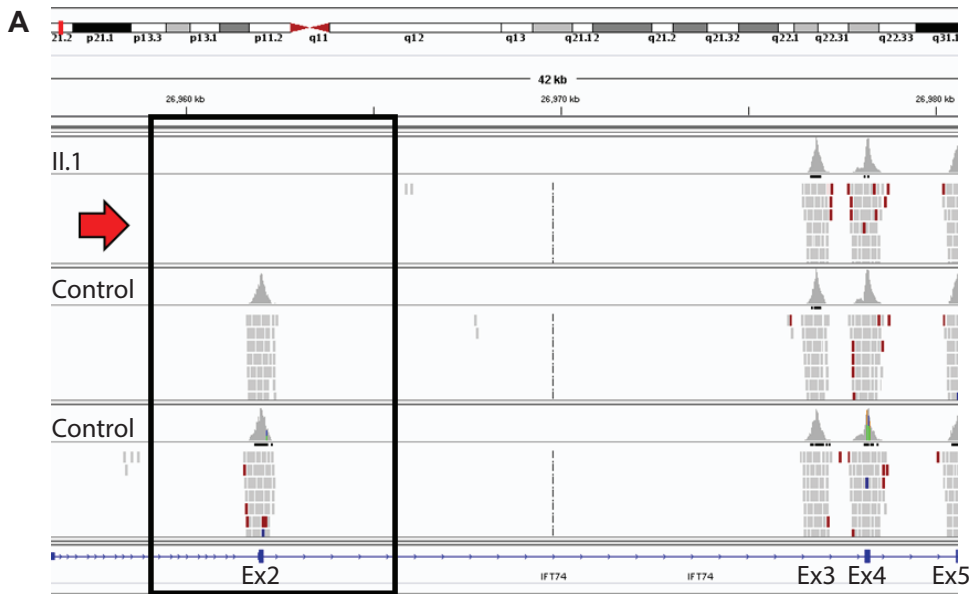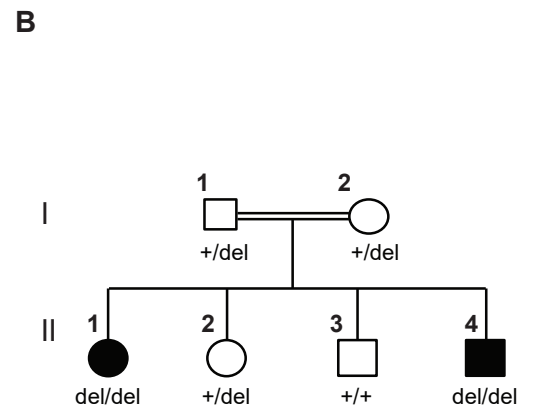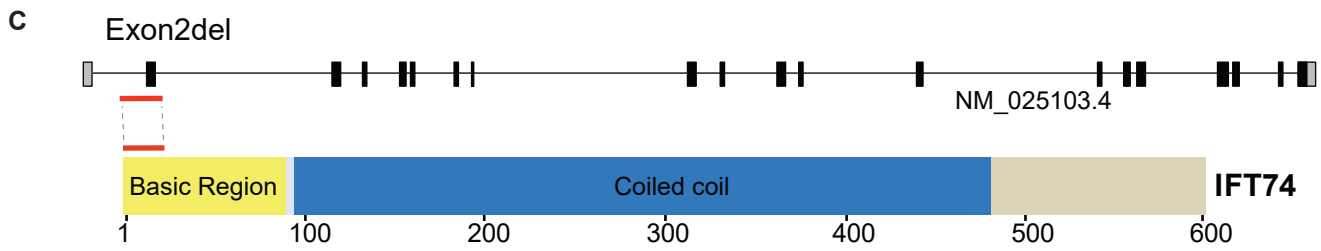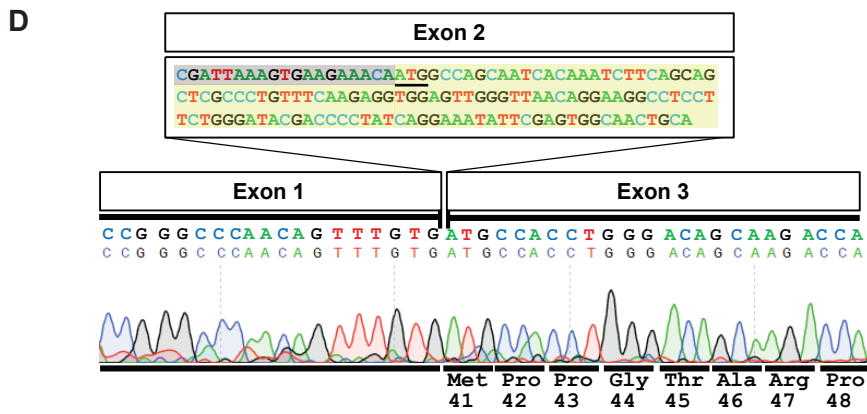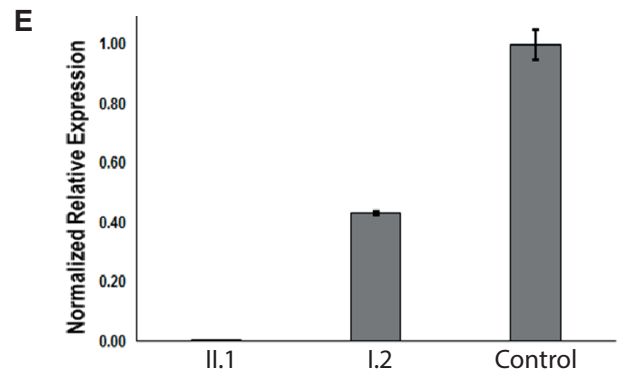

Supplement: Figure_1_revised_ddad132 [file figure_1_revised_ddad132.pdf]

**A**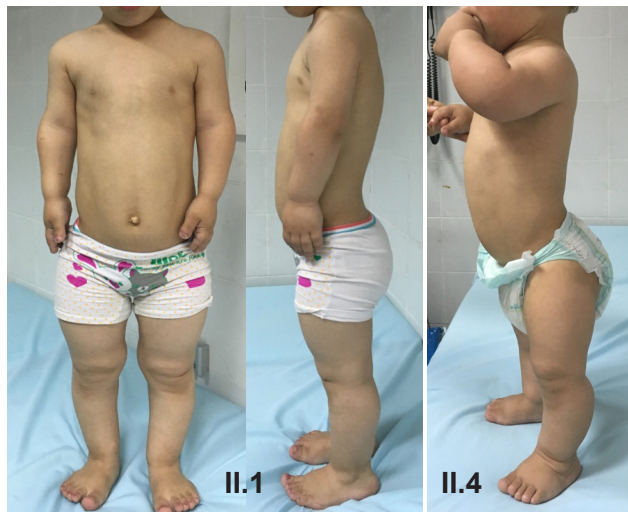**B**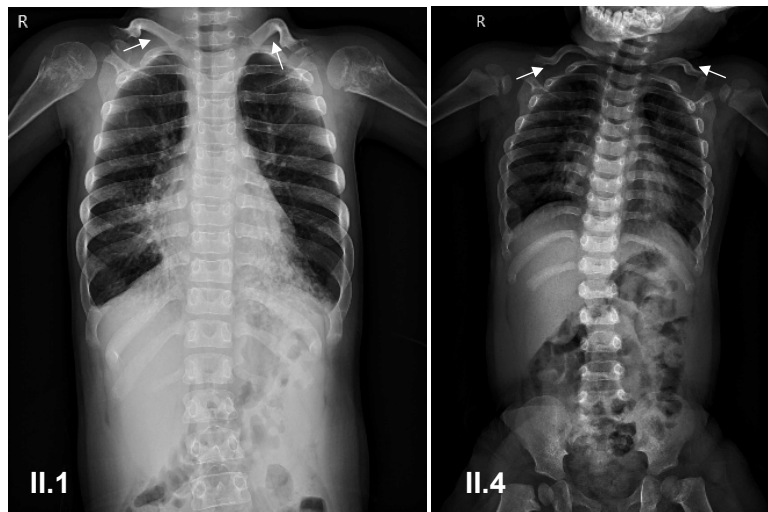**C**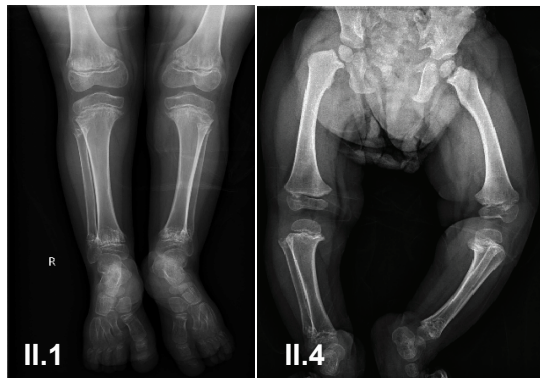**D**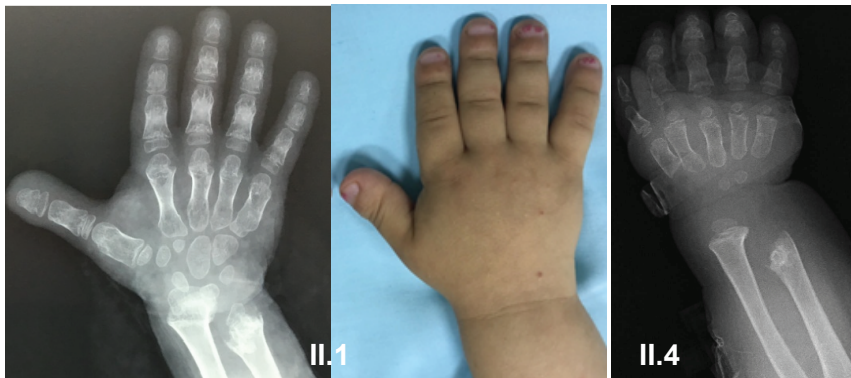**E**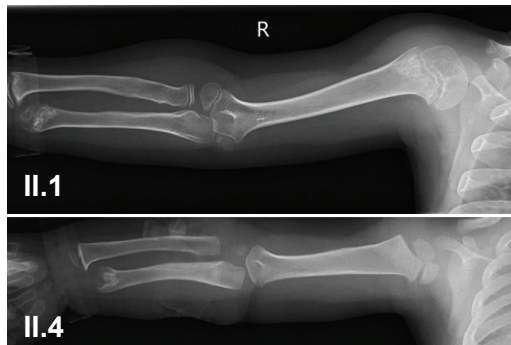**F**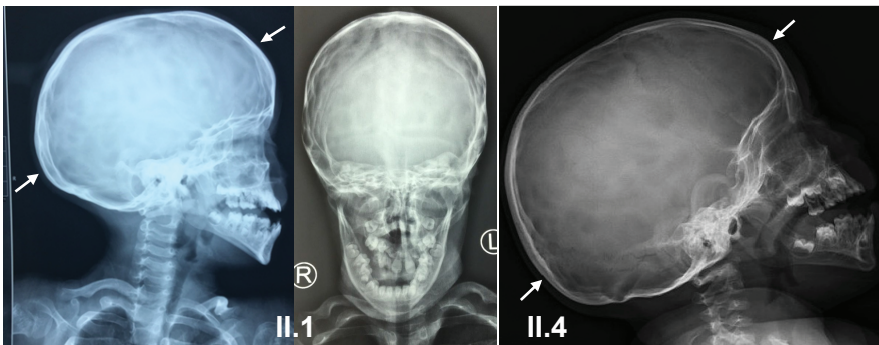**G**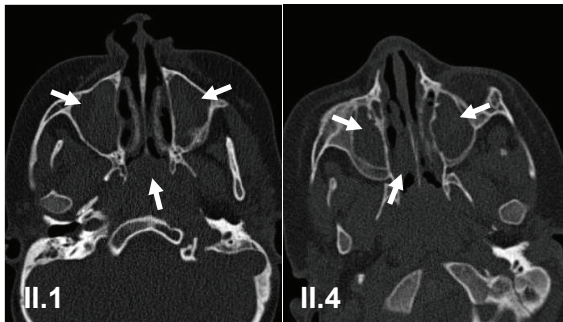**H**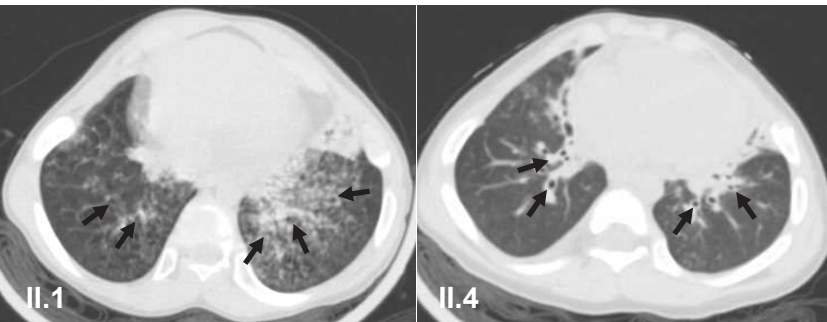

Supplement: Figure_2_revised_ddad132 [file figure_2_revised_ddad132.pdf]

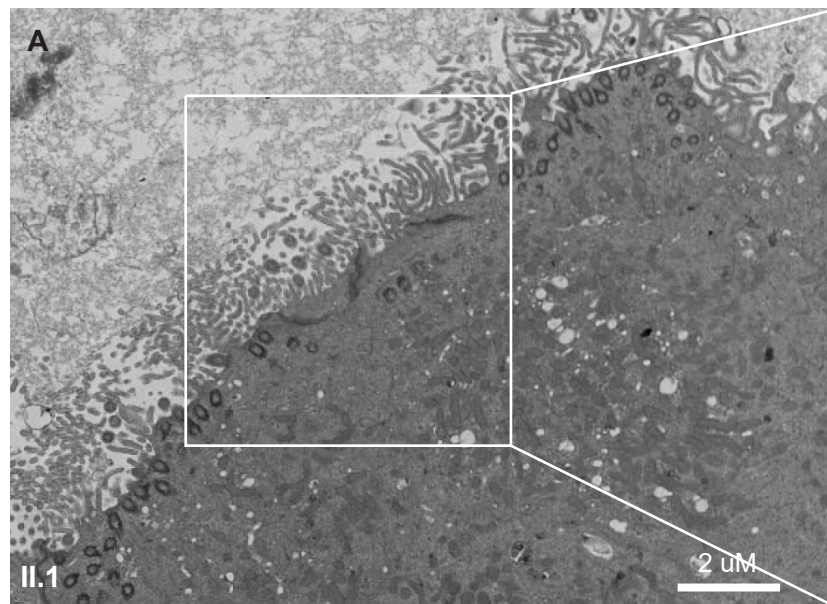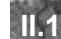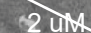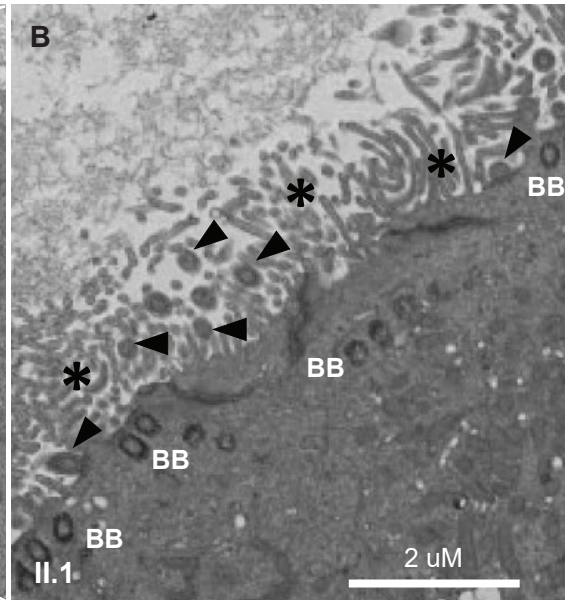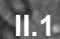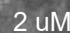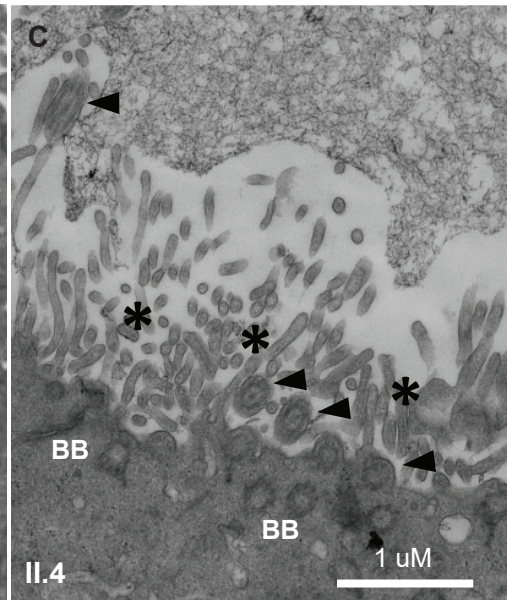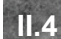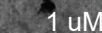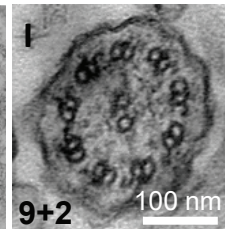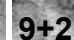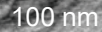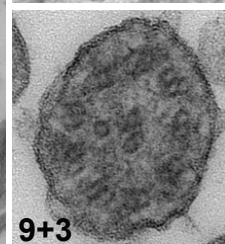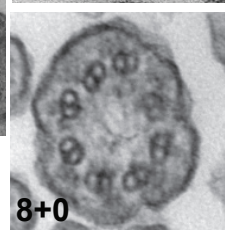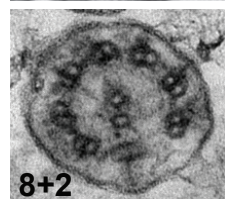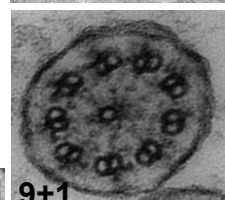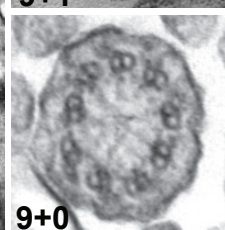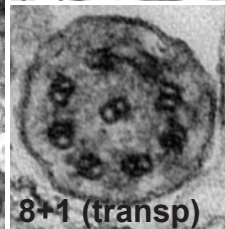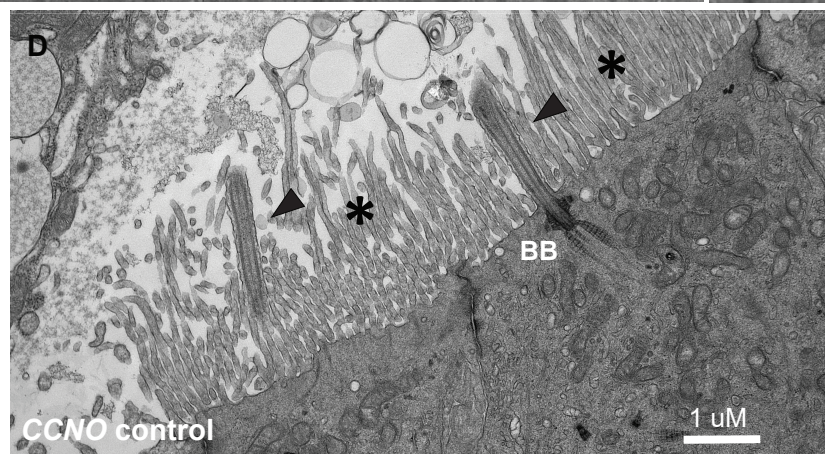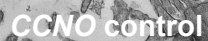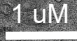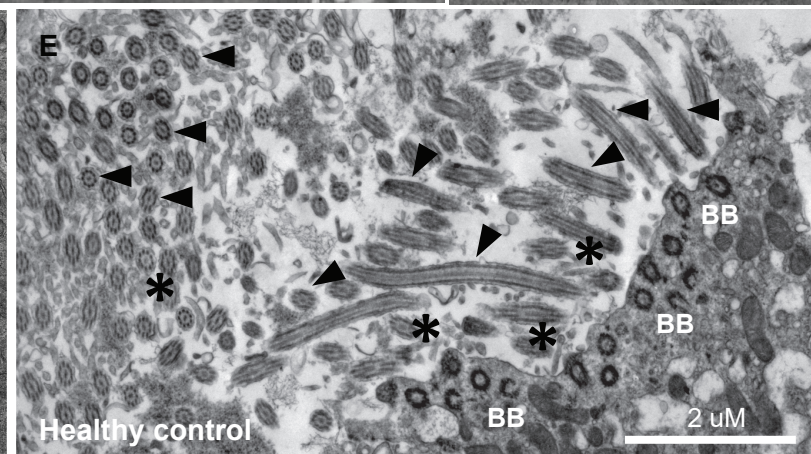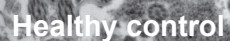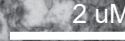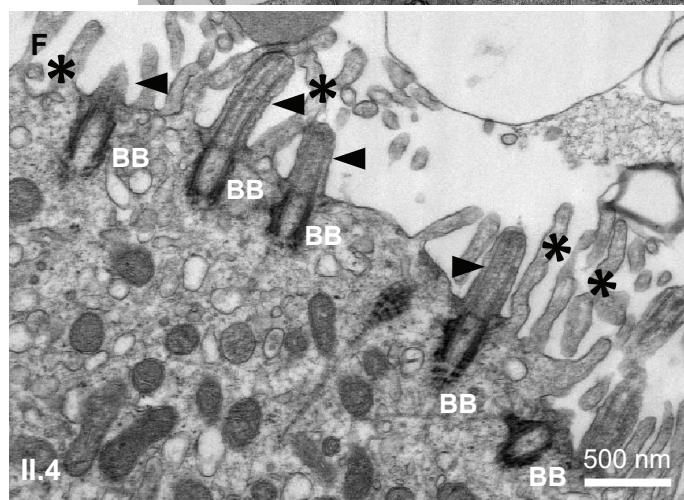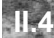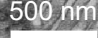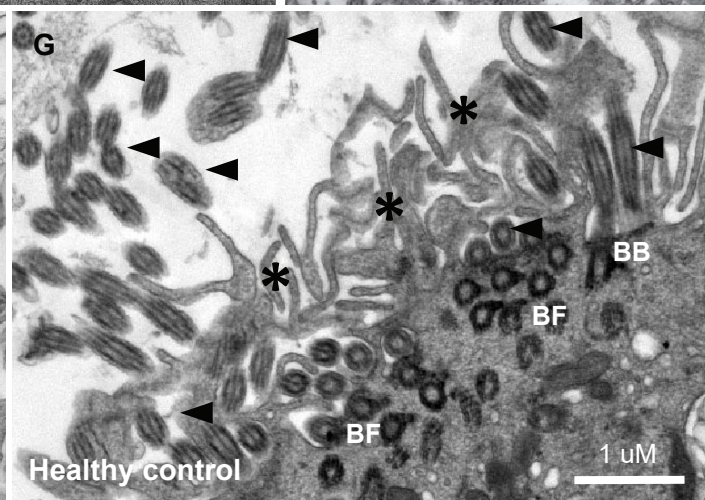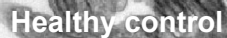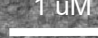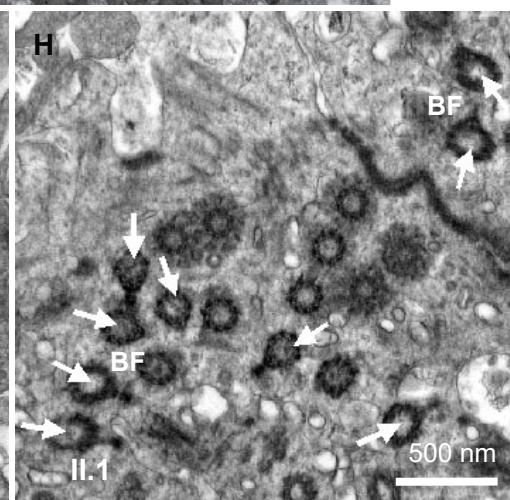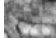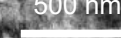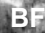

Supplement: Figure_3_revised_ddad132 [file figure_3_revised_ddad132.pdf]

A

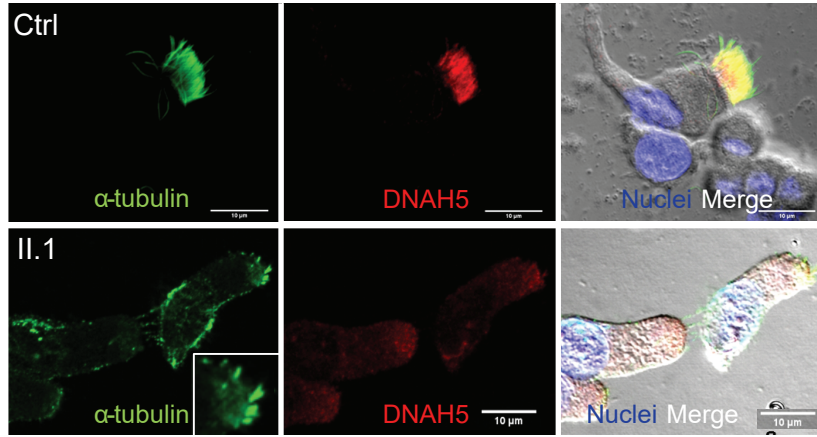

B

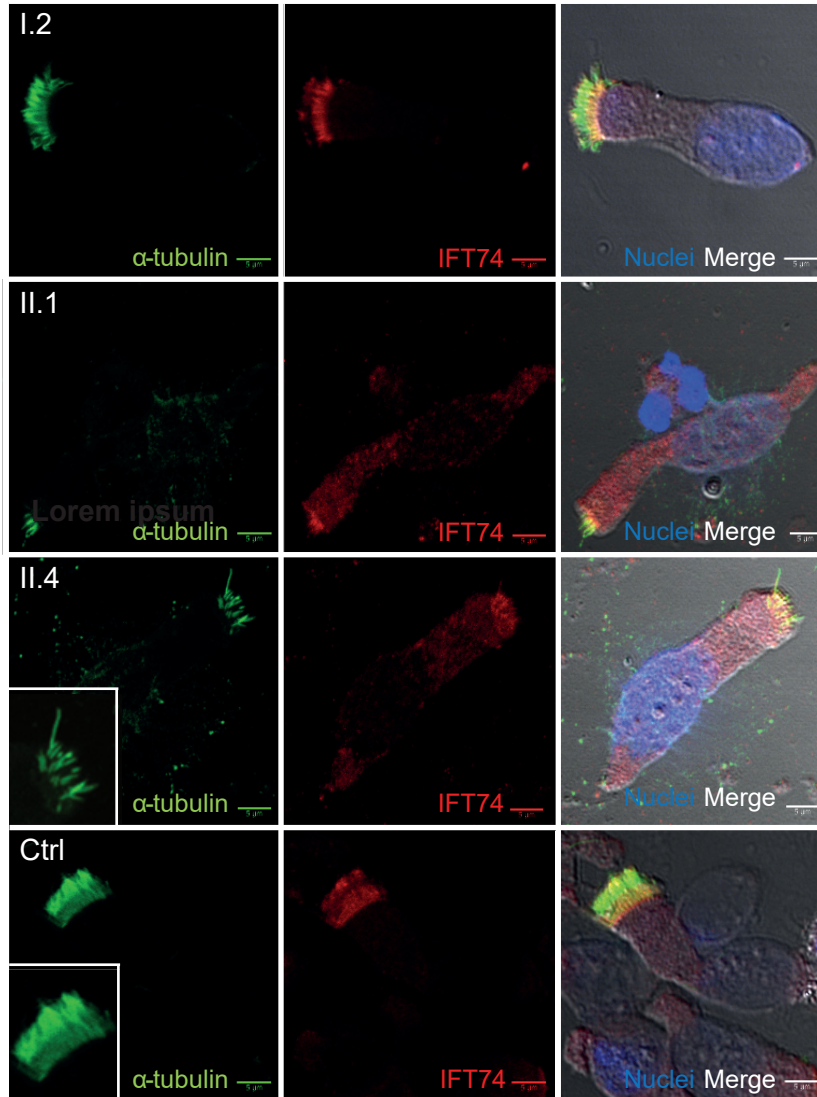

C

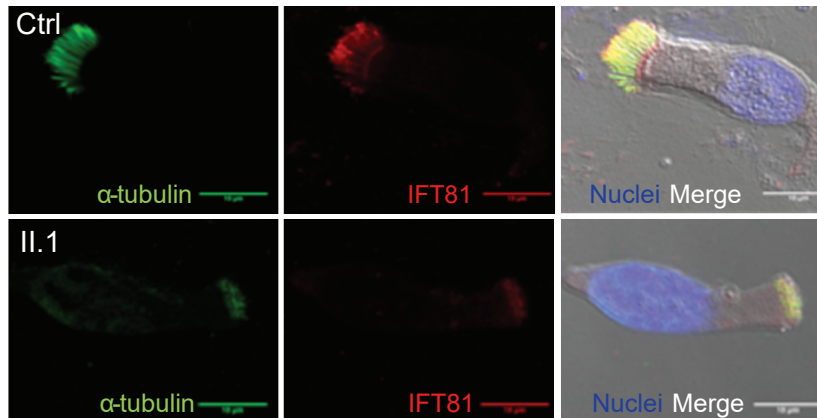

Supplement: Figure_4_ddad132 [file figure_4_ddad132.pdf]

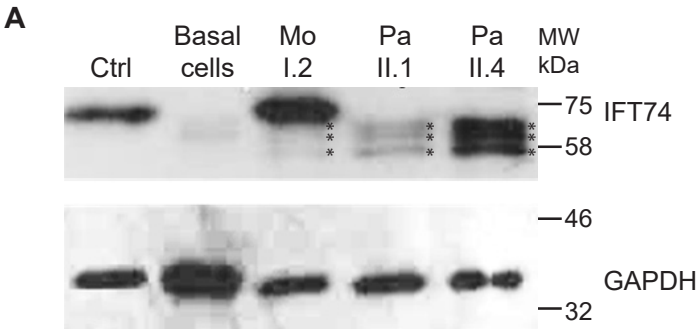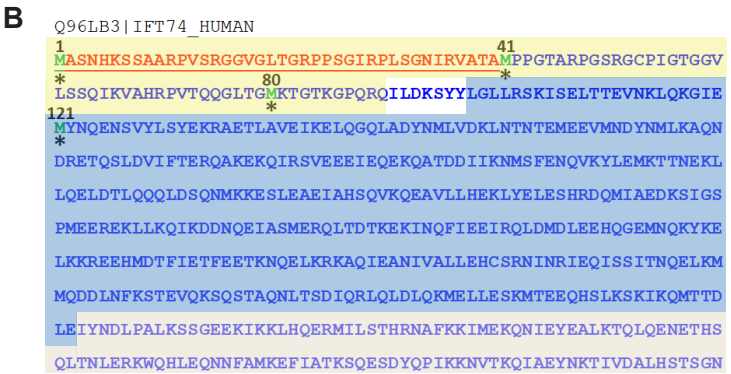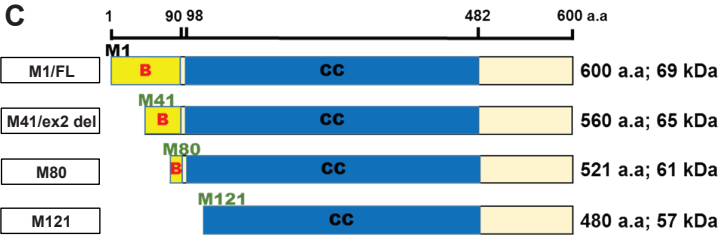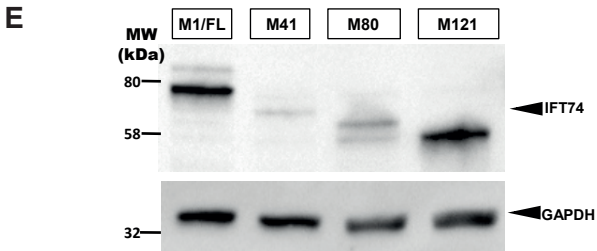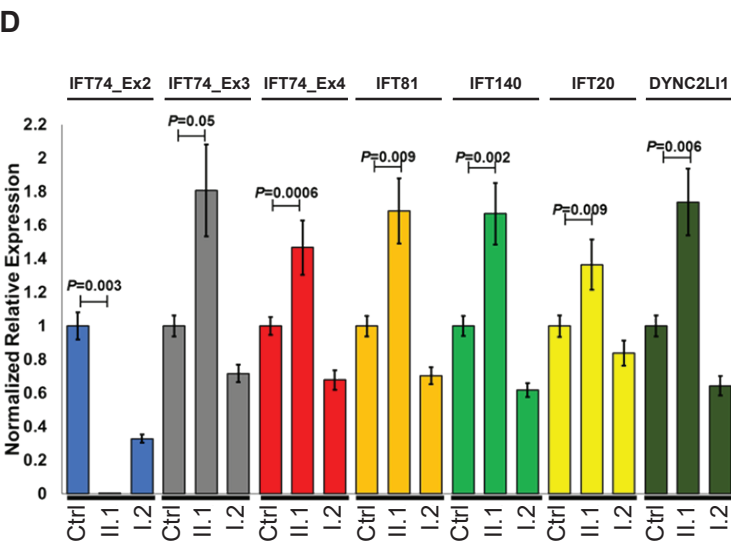

Supplement: Figure_5_revised_ddad132 [file figure_5_revised_ddad132.pdf]

A

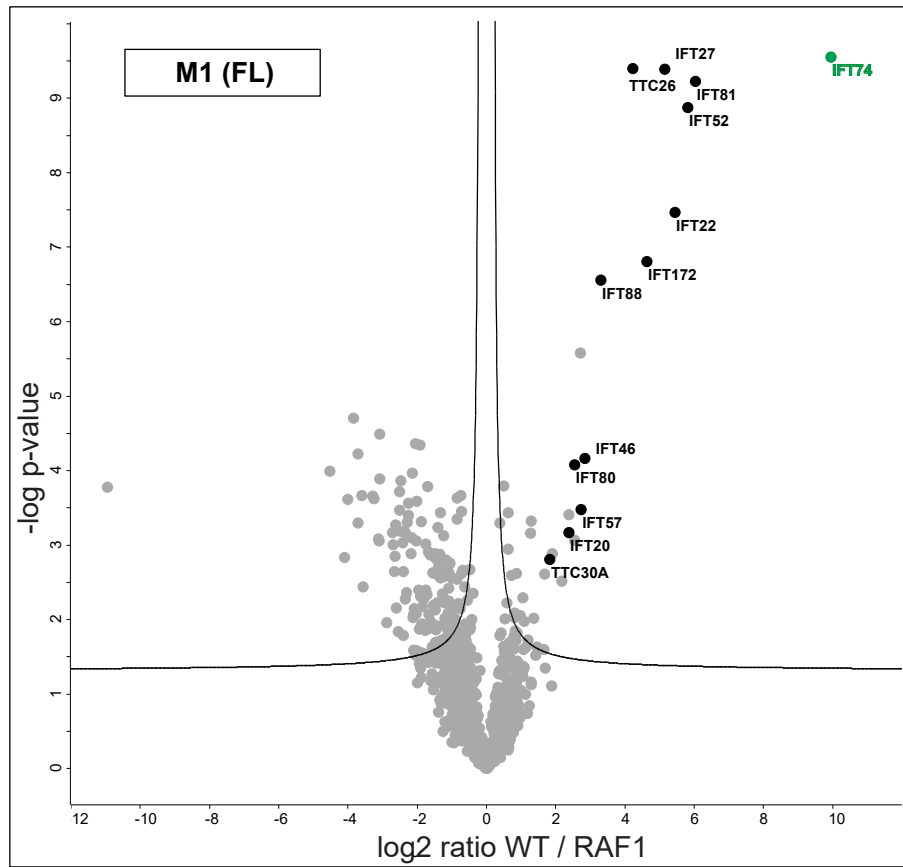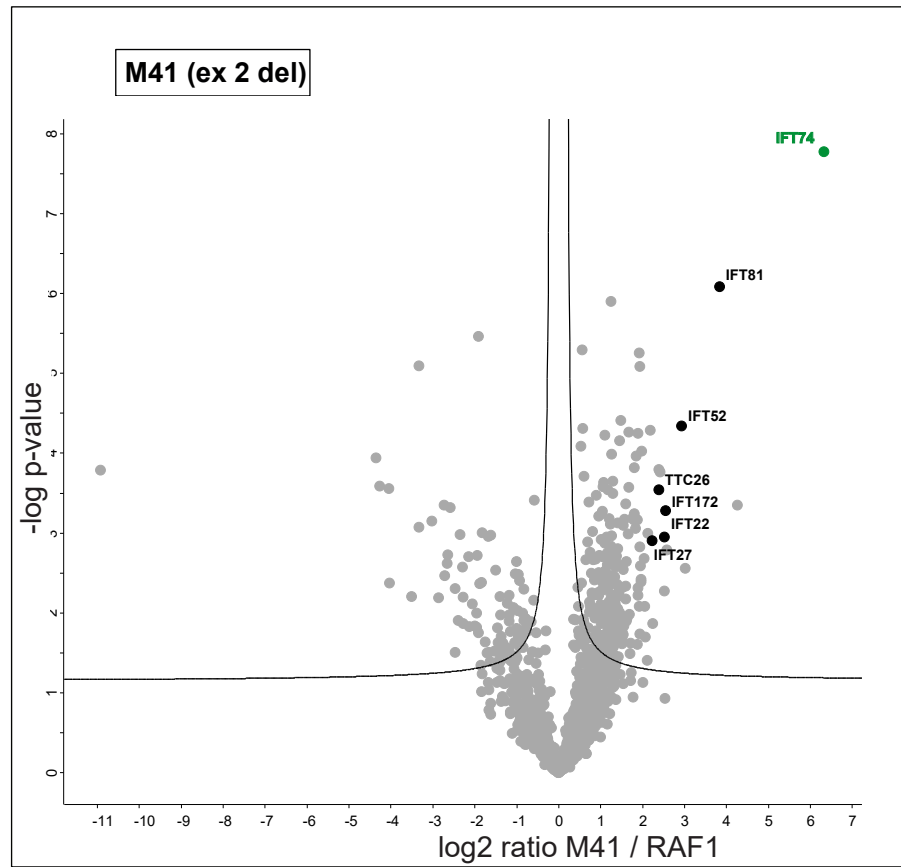

B

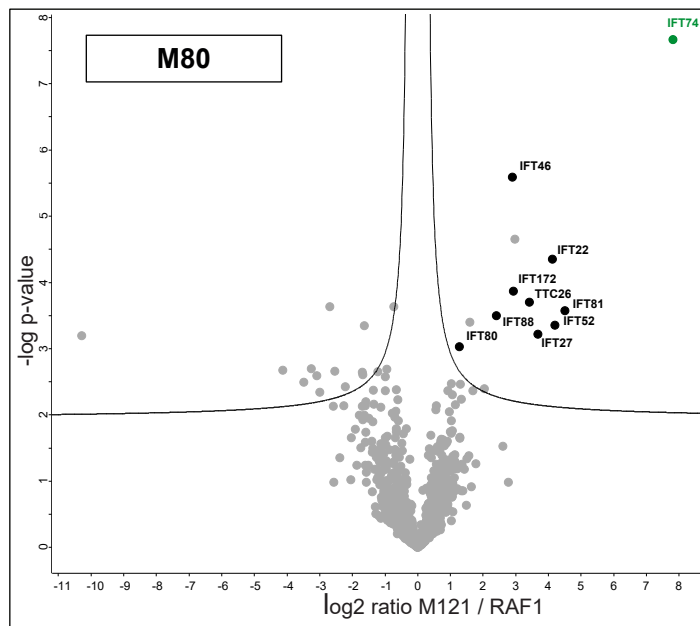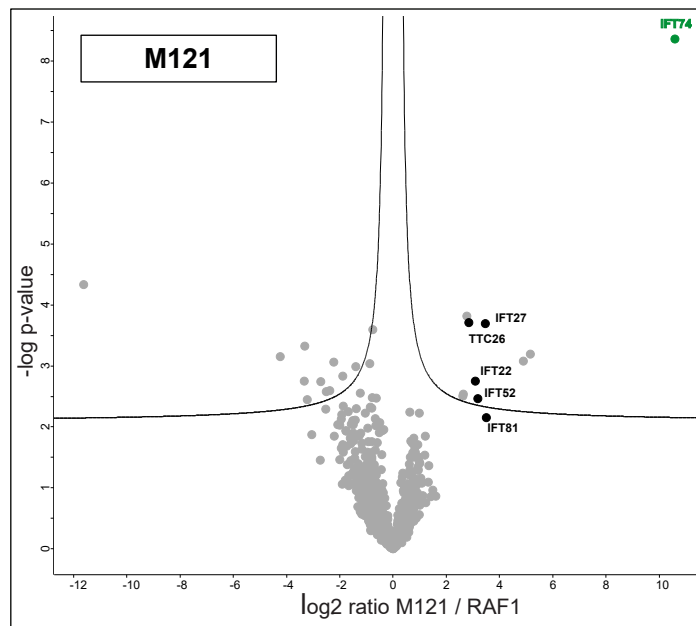

C

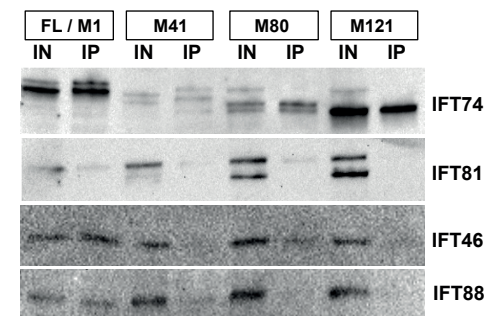

Supplement: Figure_6_ddad132 [file figure_6_ddad132.pdf]
