# Supplementary material for: Defective airway intraflagellar transport underlies a combined motile and primary ciliopathy syndrome caused by IFT74 mutations
Source: Hum Mol Genet. 2023 Aug 9;32(21):3090–104. doi: 10.1093/hmg/ddad132 (PMC10586200; doi:10.1093/hmg/ddad132)
Supplement: Supplemental_data_2nd_revision_cleaned_ddad132 [file supplemental_data_2nd_revision_cleaned_ddad132.docx]

**
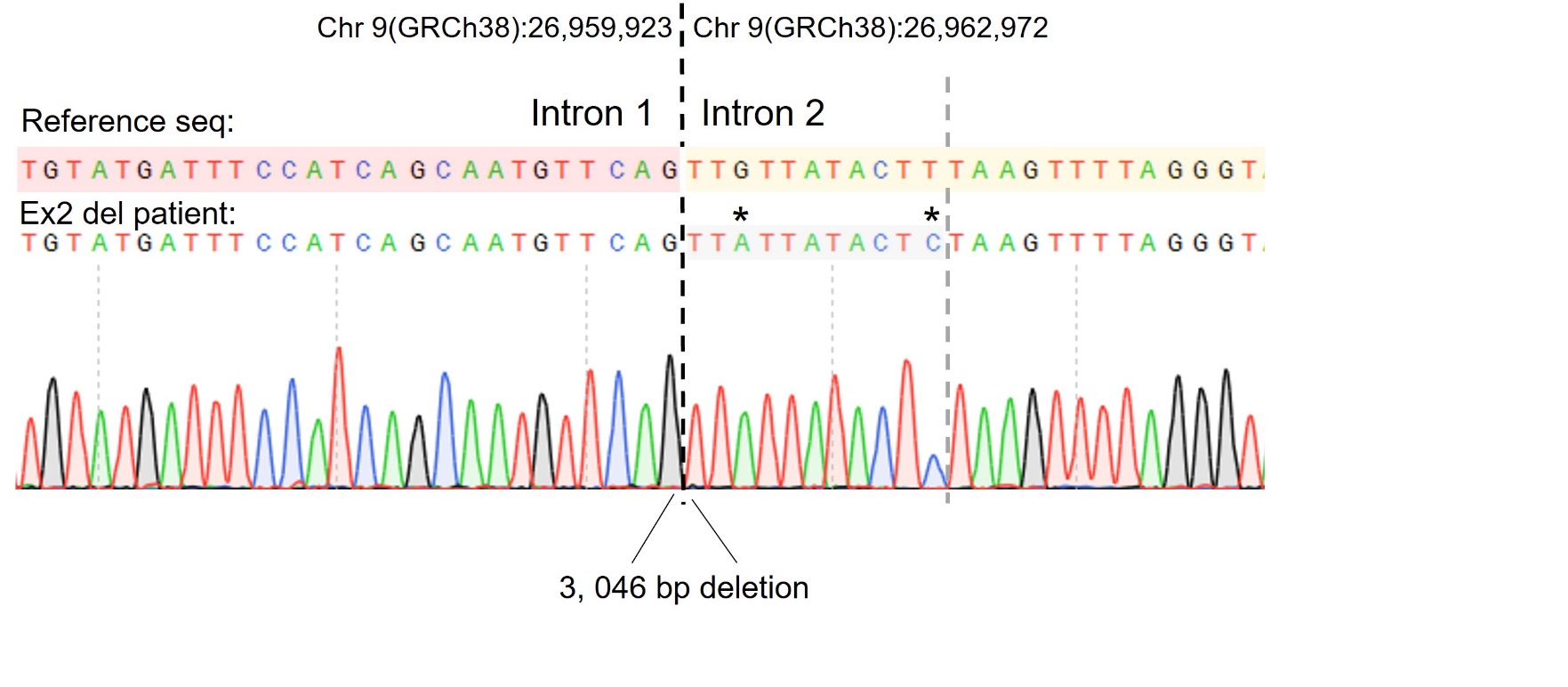
**

**Figure S1. Genomic breakpoint PCR of IFT74 exon 2 deletion amplified in affected patient II.1**

**
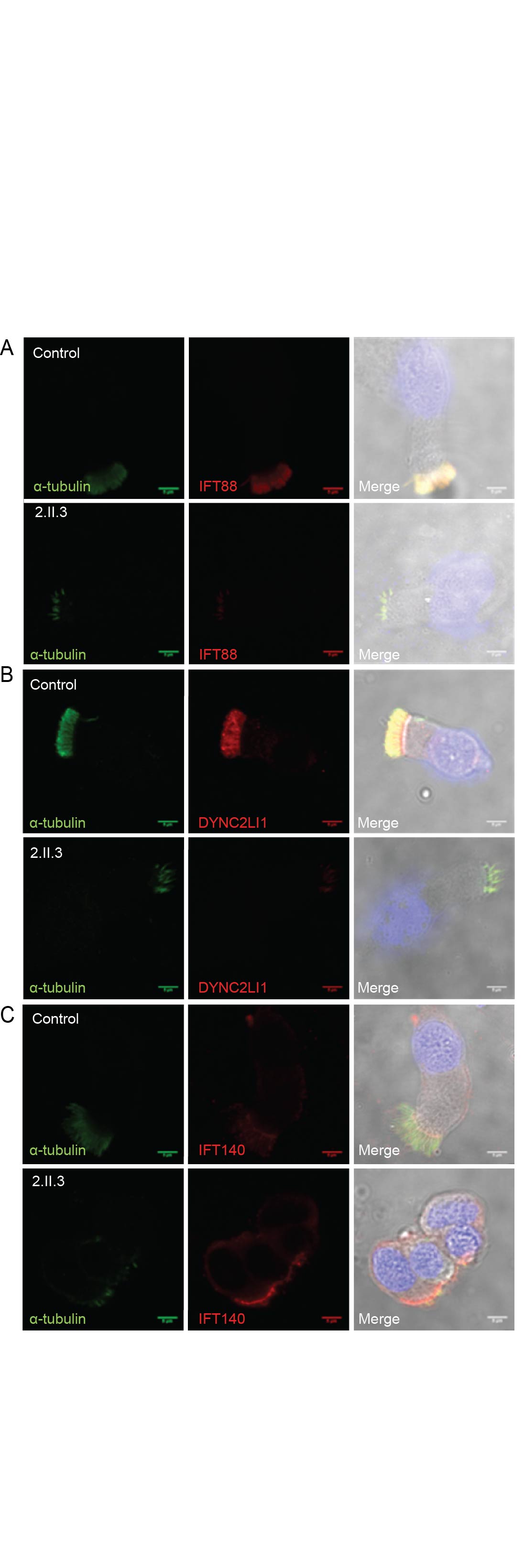
**

**Figure S2. Immunofluorescence staining in respiratory cilia from affected individual II.1 and a healthy control of intraflagellar transport components.** Cilia marker acetylated alpha tubulin (green), is co-stained with antibody markers for proteins of the IFT-B (IFT88) and IFT-A (IFT40) complex, and the retrograde IFT motor (DYNC2LI1) (all red). Nuclei stained with DAPI (blue). Scale bars, 10 um.


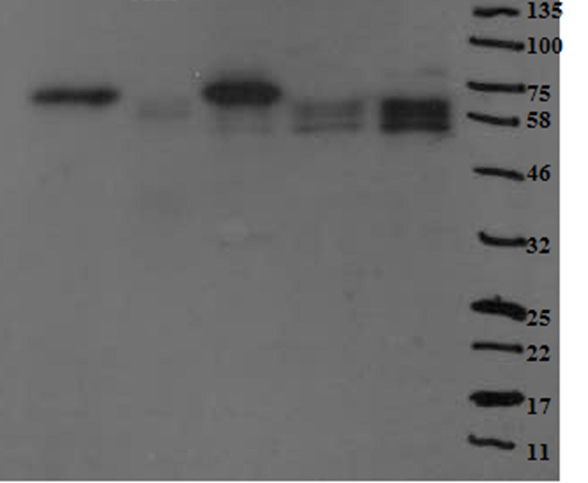


**Figure S3. Full Western blot, from Figure 5A main text.**

| **Gene ID** | **Description** | **Location** |
| --- | --- | --- |
| ACVR2B | activin A receptor type 2B | 3p22.2 |
| AGBL2 | ATP/GTP binding protein like 2 | 11p11.2 |
| AHI1 | Abelson helper integration site 1 | 6q23.3 |
| AK7 | adenylate kinase 7 | 14q32.2 |
| AK8 | adenylate kinase 8 | 9q34.13 |
| ALPK3 | alpha kinase 3 | 15q25.3 |
| ANK2 | ankyrin 2 | 4q25-q26 |
| ANK3 | ankyrin 3 | 10q21.2 |
| ANKS6 | ankyrin repeat and sterile alpha motif domain containing 6 | 9q22.33 |
| ARL13B | ADP ribosylation factor like GTPase 13B | 3q11.1-q11.2 |
| ATXN1 | ataxin 1 | 6p22.3 |
| B4GALNT3 | beta-1,4-N-acetyl-galactosaminyltransferase 3 | 12p13.33 |
| BBOF1 | basal body orientation factor 1 | 14q24.3 |
| BBS5 | Bardet-Biedl syndrome 5 | 2q31.1 |
| BTBD16 | BTB domain containing 16 | 10q26.13 |
| C11orf65 | chromosome 11 open reading frame 65 | 11q22.3 |
| C20orf96 | chromosome 20 open reading frame 96 | 20p13 |
| C21orf58 | chromosome 21 open reading frame 58 | 21q22.3 |
| C22orf23 | chromosome 22 open reading frame 23 | 22q13.1 |
| C2CD3 | C2 calcium dependent domain containing 3 | 11q13.4 |
| C3 | complement C3 | 19p13.3 |
| CAPS | calcyphosine | 19p13.3 |
| CAPSL | calcyphosine like | 5p13.2 |
| CASC1 | cancer susceptibility 1 | 12p12.1 |
| CATIP | ciliogenesis associated TTC17 interacting protein | 2q35 |
| CC2D2A | coiled-coil and C2 domain containing 2A | 4p15.32 |
| CCDC103 | coiled-coil domain containing 103 | 17q21.31 |
| CCDC105 | coiled-coil domain containing 105 | 19p13.12 |
| CCDC113 | coiled-coil domain containing 113 | 16q21 |
| CCDC170 | coiled-coil domain containing 170 | 6q25.1 |
| CCDC18 | coiled-coil domain containing 18 | 1p22.1 |
| CCDC180 | coiled-coil domain containing 180 | 9q22.33 |
| CCDC191 | coiled-coil domain containing 191 | 3q13.31 |
| CCDC24 | coiled-coil domain containing 24 | 1p34.1 |
| CCDC39 | coiled-coil domain containing 39 | 3q26.33 |
| CCDC40 | coiled-coil domain containing 40 | 17q25.3 |
| CCDC65 | coiled-coil domain containing 65 | 12q13.12 |
| CCDC74A | coiled-coil domain containing 74A | 2q21.1 |
| CCDC78 | coiled-coil domain containing 78 | 16p13.3 |
| CCDC96 | coiled-coil domain containing 96 | 4p16.1 |
| CCNO | cyclin O | 5q11.2 |
| CCP110 | centriolar coiled-coil protein 110 | 16p12.3 |
| CEP104 | centrosomal protein 104 | 1p36.32 |
| CEP120 | centrosomal protein 120 | 5q23.2 |
| CEP126 | centrosomal protein 126 | 11q22.1 |
| CEP164 | centrosomal protein 164 | 11q23.3 |
| CEP19 | centrosomal protein 19 | 3q29 |
| CEP290 | centrosomal protein 290 | 12q21.32 |
| CEP41 | centrosomal protein 41 | 7q32.2 |
| CEP83 | centrosomal protein 83 | 12q22 |
| CES1 | carboxylesterase 1 | 16q12.2 |
| CETN2 | centrin 2 | Xq28 |
| CFAP100 | cilia and flagella associated protein 100 | 3q21.3 |
| CFAP126 | cilia and flagella associated protein 126 | 1q23.3 |
| CFAP157 | cilia and flagella associated protein 157 | 9q34.11 |
| CFAP161 | cilia and flagella associated protein 161 | 15q25.1 |
| CFAP206 | cilia and flagella associated protein 206 | 6q15 |
| CFAP221 | cilia and flagella associated protein 221 | 2q14.2 |
| CFAP298 | cilia and flagella associated protein 298 | 21q22.11 |
| CFAP299 | cilia and flagella associated protein 299 | 4q21.21 |
| CFAP300 | cilia and flagella associated protein 300 | 11q22.1 |
| CFAP36 | cilia and flagella associated protein 36 | 2p16.1 |
| CFAP410 | cilia and flagella associated protein 410 | 21q22.3 |
| CFAP43 | cilia and flagella associated protein 43 | 10q25.1 |
| CFAP44 | cilia and flagella associated protein 44 | 3q13.2 |
| CFAP45 | cilia and flagella associated protein 45 | 1q23.2 |
| CFAP46 | cilia and flagella associated protein 46 | 10q26.3 |
| CFAP52 | cilia and flagella associated protein 52 | 17p13.1 |
| CFAP53 | cilia and flagella associated protein 53 | 18q21.1 |
| CFAP54 | cilia and flagella associated protein 54 | 12q23.1 |
| CFAP57 | cilia and flagella associated protein 57 | 1p34.2 |
| CFAP58 | cilia and flagella associated protein 58 | 10q25.1 |
| CFAP61 | cilia and flagella associated protein 61 | 20p11.23 |
| CFAP65 | cilia and flagella associated protein 65 | 2q35 |
| CFAP70 | cilia and flagella associated protein 70 | 10q22.2 |
| CFAP73 | cilia and flagella associated protein 73 | 12q24.13 |
| CFAP97 | cilia and flagella associated protein 97 | 4q35.1 |
| CFC1 | cripto, FRL-1, cryptic family 1 | 2q21.1 |
| COL7A1 | collagen type VII alpha 1 chain | 3p21.31 |
| CPLANE1 | ciliogenesis and planar polarity effector 1 | 5p13.2 |
| CRELD1 | cysteine rich with EGF like domains 1 | 3p25.3 |
| CSPP1 | centrosome and spindle pole associated protein 1 | 8q13.1-q13.2 |
| CYB5D1 | cytochrome b5 domain containing 1 | 17p13.1 |
| DAB1 | DAB1, reelin adaptor protein | 1p32.2 |
| DAW1 | dynein assembly factor with WD repeats 1 | 2q36.3 |
| DBH | dopamine beta-hydroxylase | 9q34.2 |
| DCDC2 | doublecortin domain containing 2 | 6p22.3 |
| DDAH1 | dimethylarginine dimethylaminohydrolase 1 | 1p22.3 |
| DDX59 | DEAD-box helicase 59 | 1q32.1 |
| DLEC1 | DLEC1, cilia and flagella associated protein | 3p22.2 |
| DNAAF1 | dynein axonemal assembly factor 1 | 16q24.1 |
| DNAAF11 | leucine rich repeat containing 6 | 8q24.22 |
| DNAAF2 | dynein axonemal assembly factor 2 | 14q21.3 |
| DNAAF3 | dynein axonemal assembly factor 3 | 19q13.42 |
| DNAAF4 | dynein axonemal assembly factor 4 | 15q21.3 |
| DNAAF5 | dynein axonemal assembly factor 5 | 7p22.3 |
| DNAAF6 | PIH1 domain containing 3 | Xq22.3 |
| DNAH1 | dynein axonemal heavy chain 1 | 3p21.1 |
| DNAH10 | dynein axonemal heavy chain 10 | 12q24.31 |
| DNAH11 | dynein axonemal heavy chain 11 | 7p15.3 |
| DNAH12 | dynein axonemal heavy chain 12 | 3p14.3 |
| DNAH14 | dynein axonemal heavy chain 14 | 1q42.12 |
| DNAH17 | dynein axonemal heavy chain 17 | 17q25.3 |
| DNAH2 | dynein axonemal heavy chain 2 | 17p13.1 |
| DNAH3 | dynein axonemal heavy chain 3 | 16p12.3 |
| DNAH5 | dynein axonemal heavy chain 5 | 5p15.2 |
| DNAH6 | dynein axonemal heavy chain 6 | 2p11.2 |
| DNAH7 | dynein axonemal heavy chain 7 | 2q32.3 |
| DNAH8 | dynein axonemal heavy chain 8 | 6p21.2 |
| DNAH9 | dynein axonemal heavy chain 9 | 17p12 |
| DNAI1 | dynein axonemal intermediate chain 1 | 9p13.3 |
| DNAI2 | dynein axonemal intermediate chain 2 | 17q25.1 |
| DNAJB13 | DnaJ heat shock protein family (Hsp40) member B13 | 11q13.4 |
| DNAL1 | dynein axonemal light chain 1 | 14q24.3 |
| DNALI1 | dynein axonemal light intermediate chain 1 | 1p34.3 |
| DPCD | deleted in primary ciliary dyskinesia homolog (mouse) | 10q24.32 |
| DRC1 | dynein regulatory complex subunit 1 | 2p23.3 |
| DRC3 | dynein regulatory complex subunit 3 | 17p11.2 |
| DRC7 | dynein regulatory complex subunit 7 | 16q21 |
| DYNC2H1 | dynein cytoplasmic 2 heavy chain 1 | 11q22.3 |
| DYNC2LI1 | dynein cytoplasmic 2 light intermediate chain 1 | 2p21 |
| DYNLL1 | dynein light chain LC8-type 1 | 12q24.31 |
| DYNLL2 | dynein light chain LC8-type 2 | 17q22 |
| DYNLRB2 | dynein light chain roadblock-type 2 | 16q23.2 |
| DYNLT1 | dynein light chain Tctex-type 1 | 6q25.3 |
| DZIP1L | DAZ interacting zinc finger protein 1 like | 3q22.3 |
| EFCAB6 | EF-hand calcium binding domain 6 | 22q13.2-q13.31 |
| EFHB | EF-hand domain family member B | 3p24.3 |
| EFHC1 | EF-hand domain containing 1 | 6p12.2 |
| EFHC2 | EF-hand domain containing 2 | Xp11.3 |
| ENKD1 | enkurin domain containing 1 | 16q22.1 |
| ENKUR | enkurin, TRPC channel interacting protein | 10p12.1 |
| EPB41L3 | erythrocyte membrane protein band 4.1 like 3 | 18p11.31 |
| EVC | EvC ciliary complex subunit 1 | 4p16.2 |
| EVC2 | EvC ciliary complex subunit 2 | 4p16.2 |
| FANK1 | fibronectin type III and ankyrin repeat domains 1 | 10q26.2 |
| FOXJ1 | forkhead box J1 | 17q25.1 |
| GALNT11 | polypeptide N-acetylgalactosaminyltransferase 11 | 7q36.1 |
| GAS8 | growth arrest specific 8 | 16q24.3 |
| GDF1 | growth differentiation factor 1 | 19p13.11 |
| GLB1L2 | galactosidase beta 1 like 2 | 11q25 |
| GLI3 | GLI family zinc finger 3 | 7p14.1 |
| GLIS2 | GLIS family zinc finger 2 | 16p13.3 |
| GLIS3 | GLIS family zinc finger 3 | 9p24.2 |
| GOLGB1 | golgin B1 | 3q13.33 |
| HNF1B | HNF1 homeobox B | 17q12 |
| HYDIN | HYDIN, axonemal central pair apparatus protein | 16q22.2 |
| IFT122 | intraflagellar transport 122 | 3q21.3-q22.1 |
| IFT140 | intraflagellar transport 140 | 16p13.3 |
| IFT172 | intraflagellar transport 172 | 2p23.3 |
| IFT22 | intraflagellar transport 22 | 7q22.1 |
| IFT27 | intraflagellar transport 27 | 22q12.3 |
| IFT43 | intraflagellar transport 43 | 14q24.3 |
| IFT57 | intraflagellar transport 57 | 3q13.12-q13.13 |
| IFT74 | intraflagellar transport 74 | 9p21.2 |
| IFT80 | intraflagellar transport 80 | 3q25.33 |
| IFT81 | intraflagellar transport 81 | 12q24.11 |
| IFT88 | intraflagellar transport 88 | 13q12.11 |
| INPP5E | inositol polyphosphate-5-phosphatase E | 9q34.3 |
| INTU | inturned planar cell polarity protein | 4q28.1 |
| INVS | inversin | 9q31.1 |
| IQCB1 | IQ motif containing B1 | 3q13.33 |
| IQCG | IQ motif containing G | 3q29 |
| IQUB | IQ motif and ubiquitin domain containing | 7q31.32 |
| KATNA1 | katanin catalytic subunit A1 | 6q25.1 |
| KATNAL2 | katanin catalytic subunit A1 like 2 | 18q21.1 |
| KIAA0586 | KIAA0586 | 14q23.1 |
| KIAA0753 | KIAA0753 | 17p13.1 |
| KIAA0754 | KIAA0754 | 1p34.2 |
| KIF17 | kinesin family member 17 | 1p36.12 |
| KIF24 | kinesin family member 24 | 9p13.3 |
| KIF27 | kinesin family member 27 | 9q21.32 |
| KIF7 | kinesin family member 7 | 15q26.1 |
| KIFAP3 | kinesin associated protein 3 | 1q24.2 |
| KNDC1 | kinase non-catalytic C-lobe domain containing 1 | 10q26.3 |
| LBR | lamin B receptor | 1q42.12 |
| LRGUK | leucine rich repeats and guanylate kinase domain containing | 7q33 |
| LRRC23 | leucine rich repeat containing 23 | 12p13.31 |
| LRRC34 | leucine rich repeat containing 34 | 3q26.2 |
| LRRC56 | leucine rich repeat containing 56 | 11p15.5 |
| LRTOMT | leucine rich transmembrane and O-methyltransferase domain containing | 11q13.4 |
| LTBP1 | latent transforming growth factor beta binding protein 1 | 2p22.3 |
| MAATS1 | MYCBP associated and testis expressed 1 | 3q13.33 |
| MAK | male germ cell associated kinase | 6p24.2 |
| MAPK10 | mitogen-activated protein kinase 10 | 4q21.3 |
| MAPK15 | mitogen-activated protein kinase 15 | 8q24.3 |
| MAPRE3 | microtubule associated protein RP/EB family member 3 | 2p23.3 |
| MASP1 | mannan binding lectin serine peptidase 1 | 3q27.3 |
| MCIDAS | multiciliate differentiation and DNA synthesis associated cell cycle protein | 5q11.2 |
| MDH1B | malate dehydrogenase 1B | 2q33.3 |
| MKS1 | Meckel syndrome, type 1 | 17q22 |
| MLF1 | myeloid leukemia factor 1 | 3q25.32 |
| MNS1 | meiosis specific nuclear structural 1 | 15q21.3 |
| MORN2 | MORN repeat containing 2 | 2p22.1 |
| MORN3 | MORN repeat containing 3 | 12q24.31 |
| MPDZ | multiple PDZ domain crumbs cell polarity complex component | 9p23 |
| MTTP | microsomal triglyceride transfer protein | 4q23 |
| MUC1 | mucin 1, cell surface associated | 1q22 |
| MUSTN1 | musculoskeletal, embryonic nuclear protein 1 | 3p21.1 |
| NCALD | neurocalcin delta | 8q22.3 |
| NEK1 | NIMA related kinase 1 | 4q33 |
| NEK11 | NIMA related kinase 11 | 3q22.1 |
| NEK2 | NIMA related kinase 2 | 1q32.3 |
| NEK3 | NIMA related kinase 3 | 13q14.3 |
| NEK8 | NIMA related kinase 8 | 17q11.1 |
| NME5 | NME/NM23 family member 5 | 5q31.2 |
| NME7 | NME/NM23 family member 7 | 1q24.2 |
| NME8 | NME/NM23 family member 8 | 7p14.1 |
| NODAL | nodal growth differentiation factor | 10q22.1 |
| NPHP1 | nephrocystin 1 | 2q13 |
| NPHP3 | nephrocystin 3 | 3q22.1 |
| NPHP4 | nephrocystin 4 | 1p36.31 |
| NUP188 | nucleoporin 188 | 9q34.11 |
| ODAD1 | coiled-coil domain containing 114 | 19q13.33 |
| ODAD2 | armadillo repeat containing 4 | 10p12.1 |
| ODAD3 | coiled-coil domain containing 151 | 19p13.2 |
| ODAD4 | tetratricopeptide repeat domain 25 | 17q21.2 |
| ODF2 | outer dense fiber of sperm tails 2 | 9q34.11 |
| OFD1 | OFD1, centriole and centriolar satellite protein | Xp22.2 |
| OSBPL3 | oxysterol binding protein like 3 | 7p15.3 |
| PACRG | parkin coregulated | 6q26 |
| PCNT | pericentrin | 21q22.3 |
| PDE6D | phosphodiesterase 6D | 2q37.1 |
| PIBF1 | progesterone immunomodulatory binding factor 1 | 13q21.33-q22.1 |
| PIFO | primary cilia formation | 1p13.2 |
| PIH1D1 | PIH1 domain containing 1 | 19q13.33 |
| PIH1D2 | PIH1 domain containing 2 | 11q23.1 |
| PLEKHB1 | pleckstrin homology domain containing B1 | 11q13.4 |
| POC1B | POC1 centriolar protein B | 12q21.33 |
| PPP1R42 | protein phosphatase 1 regulatory subunit 42 | 8q13.1 |
| PROM1 | prominin 1 | 4p15.32 |
| RAB36 | RAB36, member RAS oncogene family | 22q11.23 |
| RABL2A | RAB, member of RAS oncogene family like 2A | 2q14.1 |
| RABL2B | RAB, member of RAS oncogene family like 2B | 22q13.33 |
| RFX2 | regulatory factor X2 | 19p13.3 |
| RFX3 | regulatory factor X3 | 9p24.2 |
| ROCK2 | Rho associated coiled-coil containing protein kinase 2 | 2p25.1 |
| ROPN1L | rhophilin associated tail protein 1 like | 5p15.2 |
| RPGR | retinitis pigmentosa GTPase regulator | Xp11.4 |
| RPGRIP1L | RPGRIP1 like | 16q12.2 |
| RSPH1 | radial spoke head component 1 | 21q22.3 |
| RSPH10B | radial spoke head 10 homolog B | 7p22.1 |
| RSPH10B2 | radial spoke head 10 homolog B2 | 7p22.1 |
| RSPH14 | radial spoke head 14 homolog | 22q11.22-q11.23 |
| RSPH3 | radial spoke head 3 homolog | 6q25.3 |
| RSPH4A | radial spoke head component 4A | 6q22.1 |
| RSPH6A | radial spoke head 6 homolog A | 19q13.3 |
| RSPH9 | radial spoke head 9 homolog | 6p21.1 |
| RUVBL1 | RuvB like AAA ATPase 1 | 3q21.3 |
| RUVBL2 | RuvB like AAA ATPase 2 | 19q13.33 |
| SAXO2 | stabilizer of axonemal microtubules 2 | 15q25.2 |
| SBDS | SBDS, ribosome maturation factor | 7q11.21 |
| SDCCAG8 | serologically defined colon cancer antigen 8 | 1q43-q44 |
| SLC16A7 | solute carrier family 16 member 7 | 12q14.1 |
| SMC1A | structural maintenance of chromosomes 1A | Xp11.22 |
| SMYD2 | SET and MYND domain containing 2 | 1q32.3 |
| SPA17 | sperm autoantigenic protein 17 | 11q24.2 |
| SPAG1 | sperm associated antigen 1 | 8q22.2 |
| SPAG16 | sperm associated antigen 16 | 2q34 |
| SPAG17 | sperm associated antigen 17 | 1p12 |
| SPAG6 | sperm associated antigen 6 | 10p12.2 |
| SPAG8 | sperm associated antigen 8 | 9p13.3 |
| SPAG9 | sperm associated antigen 9 | 17q21.33 |
| SPATA17 | spermatogenesis associated 17 | 1q41 |
| SPATA18 | spermatogenesis associated 18 | 4q12 |
| SPATA7 | spermatogenesis associated 7 | 14q31.3 |
| SPEF1 | sperm flagellar 1 | 20p13 |
| SPEF2 | sperm flagellar 2 | 5p13.2 |
| STK36 | serine/threonine kinase 36 | 2q35 |
| STOML3 | stomatin like 3 | 13q13.2 |
| TAPT1 | transmembrane anterior posterior transformation 1 | 4p15.32 |
| TBC1D32 | TBC1 domain family member 32 | 6q22.31 |
| TCHH | trichohyalin | 1q21.3 |
| TCTE1 | t-complex-associated-testis-expressed 1 | 6p21.1 |
| TCTEX1D1 | Tctex1 domain containing 1 | 1p31.3 |
| TCTEX1D2 | Tctex1 domain containing 2 | 3q29 |
| TCTN1 | tectonic family member 1 | 12q24.11 |
| TCTN2 | tectonic family member 2 | 12q24.31 |
| TCTN3 | tectonic family member 3 | 10q24.1 |
| TEKT1 | tektin 1 | 17p13.1 |
| TEKT2 | tektin 2 | 1p34.3 |
| TEKT4 | tektin 4 | 2q11.1 |
| TMC5 | transmembrane channel like 5 | 16p12.3 |
| TMEM107 | transmembrane protein 107 | 17p13.1 |
| TMEM138 | transmembrane protein 138 | 11q12.2 |
| TMEM216 | transmembrane protein 216 | 11q13.1 |
| TMEM231 | transmembrane protein 231 | 16q23.1 |
| TMEM237 | transmembrane protein 237 | 2q33.1 |
| TMEM67 | transmembrane protein 67 | 8q22.1 |
| TOB2 | transducer of ERBB2, 2 | 22q13.2 |
| TPPP3 | tubulin polymerization promoting protein family member 3 | 16q22.1 |
| TSNAXIP1 | translin associated factor X interacting protein 1 | 16q22.1 |
| TTC12 | tetratricopeptide repeat domain 12 | 11q23.2 |
| TTC21A | tetratricopeptide repeat domain 21A | 3p22.2 |
| TTC21B | tetratricopeptide repeat domain 21B | 2q24.3 |
| TTC26 | tetratricopeptide repeat domain 26 | 7q34 |
| TTC28 | tetratricopeptide repeat domain 28 | 22q12.1 |
| TTC29 | tetratricopeptide repeat domain 29 | 4q31.22 |
| TTC30B | tetratricopeptide repeat domain 30B | 2q31.2 |
| TTLL1 | tubulin tyrosine ligase like 1 | 22q13.2 |
| TTLL3 | tubulin tyrosine ligase like 3 | 3p25.3 |
| TTLL9 | tubulin tyrosine ligase like 9 | 20q11 |
| TUBA1A | tubulin alpha 1a | 12q13.12 |
| TUBB4B | tubulin beta 4B class IVb | 9q34.3 |
| ULK4 | unc-51 like kinase 4 | 3p22.1 |
| UMOD | uromodulin | 16p12.3 |
| WDR1 | WD repeat domain 1 | 4p16.1 |
| WDR19 | WD repeat domain 19 | 4p14 |
| WDR34 | WD repeat domain 34 | 9q34.11 |
| WDR35 | WD repeat domain 35 | 2p24.1 |
| WDR38 | WD repeat domain 38 | 9q33.3 |
| WDR60 | WD repeat domain 60 | 7q36.3 |
| WDR63 | WD repeat domain 63 | 1p22.3 |
| WDR66 | WD repeat domain 66 | 12q24.31 |
| WDR78 | WD repeat domain 78 | 1p31.3 |
| WDR92 | WD repeat domain 92 | 2p14 |
| ZBBX | zinc finger B-box domain containing | 3q26.1 |
| ZIC3 | Zic family member 3 | Xq26.3 |
| ZMYND10 | zinc finger MYND-type containing 10 | 3p21.31 |
| ZMYND12 | zinc finger MYND-type containing 12 | 1p34.2 |
| ZNF423 | zinc finger protein 423 | 16q12.1 |

**Table S1. Panel of 321 genes used in analysis**

| **Primer ID** | **(5’- Sequence -3’)** |
| --- | --- |
| **RT-PCR of IFT74** | |
| Fwd. | CCTGCTGAGCAAGAGAGAAAGA |
| Rev. | TGTTGTAGTCTGCTAGTTGTCCT |
| **RT-PCR of GAPDH** | |
| Fwd. | GTCATCCCTGAGCTGAACGG |
| Rev. | AAGTGGTCGTTGAGGGCAAT |
| **IFT74 genomic deletion breakpoint sequencing** | |
| Fwd. | TTTGCAGTACCCCAGTCTGT |
| Rev. | GAGAGGAGGGATAGCATCGG |
| **RT-qPCR of IFT74 exon 2** | |
| Fwd. | CCTTCTGGGATACGACCCCT |
| Rev. | ACGAGAACCTGGTCTTGCTG |
| **RT-qPCR of IFT74 exon 3** | |
| Fwd. | TCCCATAGGGACTGGTGGAG |
| Rev. | GGGACCTTTCGTCCCAGTTT |
| **RT-qPCR of IFT74 exon 4** | |
| Fwd. | AAAGGTCCCCAGAGGCAAAT |
| Rev. | CAGCTAAAGTCTCAGCCCTCT |
| **RT-qPCR of GAPDH** | |
| Fwd. | TGCACCACCAACTGCTTAGC |
| Rev. | GGCATGGACTGTGGTCATGAG |
| **RT-qPCR of DYNC2LI1** | |
| Fwd. | AAGATATCAAGGACCCTGCGAG |
| Rev. | CTTTTGTACTGTTCCAGTTCCAGAT |
| **RT-qPCR of IFT140** | |
| Fwd. | GGGGTTCCAGGAAGAACCTG |
| Rev. | ACACATTCAGCAGACTCGGG |
| **RT-qPCR of IFT81** | |
| Fwd. | TCAGACCCGTGAATTTGATGGT |
| Rev. | GTCCTCTGCAAAAGACCGAA |
| **RT-qPCR of IFT20** | |
| Fwd. | CTCCTGACTGCCACTGTCAC |
| Rev. | TGGGTAACCTCTGGGTCCAA |
| **RT-qPCR of TUBA4A** | |
| Fwd. | GACAAGACCATTGGTGGAGGG |
| Rev. | GGTATGGGCCATTTCGGATCT |

**Table S3. Primer sequence used in the study**

| **Protein** | **Antibody** | **Host species** | **Source** | **Application** |
| --- | --- | --- | --- | --- |
| Acetylated tubulin | YF488 | Mouse | Proteintech | IF (1:500) |
| DNAH5 | HPA037470 | Rabbit | Sigma-Aldrich | IF (1:800) |
| IFT74 | 27334-1-AP | Rabbit | Proteintech | IF (1:500)  IB (1:1500) |
| IFT81 | 11744-1-AP | Rabbit | Proteintech | IF (1:200)  IB (1:500) |
| IFT88 | 13967-1-AP | Rabbit | Proteintech | IF (1:200)  IB (1:300) |
| DYNC2LI1 | 15949-1-AP | Rabbit | Proteintech | IF (1:300) |
| IFT46 | HPA037909 | Rabbit | Cambridge Bioscience | IB (1:100) |
| FLAG | F1804 | Mouse | Sigma-Aldrich | IB (1:1000) |
| GAPDH | G8795 | Mouse | Sigma-Aldrich | IB (1:1000) |

**Table S4. Antibodies used in immunostaining (IF) and immunoblotting (IB) of human cells**

| **Clinical spectrum** | **Reference** | **#cases** | **IFT74 allele 1** | **IFT74 allele 2** | **Consequence** |
| --- | --- | --- | --- | --- | --- |
| Bardet Biedl syndrome | Lindstrand (1) | 1 | c.1685–1G>T  (exon 20) | Exon 14-19 del  (exon 14-19) | Splice + C-terminus deletion |
| Bardet Biedl syndrome | Kleinendorst (2) | 1 | c.1685–1G>T  (exon 20) | c.371-372del (p.Gln124Argfs*9)  (exon 5) | Splice + frameshift |
| Bardet Biedl syndrome | Mardy (3) | 1 | c.1685–1G>T  (exon 20) | c.1685–1G>T  (exon 20) | Splice - homozygous |
|  |  |  |  |  |  |
| Joubert syndrome | Luo (4) | 2 | c.535C>G (p.Gln179Glu)  (exon 8) | c.92delT (p.Leu31Hisfs*25)  (exon 2) | Missense + frameshift |
|  |  | 1 | c.535C>G (p.Gln179Glu)  (exon 8) | c.306-24A>G  (exon 4) | Missense + splice |
|  |  | 1 | c.535C>G (p.Gln179Glu)  (exon 8) | c.85C>T (p.Arg29*)  (exon 2) | Missense + stop codon |
| Joubert syndrome | Zhongling (5) | 1 | c.535C>G (p.Gln179Glu)  (exon 8) | c.853G>T (p.Glu285*)  (exon 11) | Missense + stop codon |
|  |  |  |  |  |  |
| Sperm defects | Lores (6) | 2 | c.256G>A (p.Gly86Ser)  (exon 3) | c.256G>A (p.Gly86Ser)  (exon 3) | Missense (homozygous) |
|  |  |  |  |  |  |
| Asphyxiating thoracic dystrophy/CED | Hammarsjö (7) | 1 | Exon 2 del  (exon 2) | Exon 2 del  (exon 2) | Inframe loss of N-terminal 40 amino acids - homozygous |
| Asphyxiating thoracic dystrophy/CED | This study | 2 | Exon 2 del  (exon 2) | Exon 2 del  (exon 2) | Inframe loss of N-terminal 40 amino acids - homozygous |

**Table S5. Summary of phenotypes connected to previously published IFT74 mutations. References below.**

1 Lindstrand, A., Frangakis, S., Carvalho, C.M.B., Richardson, E.B., McFadden, K.A., Willer, J.R., Pehlivan, D., Liu, P.F., Pediaditakis, I.L., Sabo, A. *et al.* (2016) Copy-Number Variation Contributes to the Mutational Load of Bardet-Biedl Syndrome. *American Journal of Human Genetics*, **99**, 318-336.

2 Kleinendorst, L., Alsters, S.I.M., Abawi, O., Waisfisz, Q., Boon, E.M.J., van den Akker, E.L.T. and van Haelst, M.M. (2020) Second case of Bardet-Biedl syndrome caused by biallelic variants in IFT74. *Eur J Hum Genet*, **28**, 943-946.

3 Mardy, A.H., Hodoglugil, U., Yip, T. and Slavotinek, A.M. (2021) Third case of Bardet-Biedl syndrome caused by a biallelic variant predicted to affect splicing of IFT74. *Clin Genet*, **100**, 93-99.

4 Luo, M., Lin, Z., Zhu, T., Jin, M., Meng, D., He, R., Cao, Z., Shen, Y., Lu, C., Cai, R. *et al.* (2021) Disrupted intraflagellar transport due to IFT74 variants causes Joubert syndrome. *Genet Med*, **23**, 1041-1049.

5 Zhongling, K., Guoming, L., Yanhui, C. and Xiaoru, C. (2021) Case Report: Second Report of Joubert Syndrome Caused by Biallelic Variants in IFT74. *Front Genet*, **12**, 738157.

6 Lores, P., Kherraf, Z.E., Amiri-Yekta, A., Whitfield, M., Daneshipour, A., Stouvenel, L., Cazin, C., Cavarocchi, E., Coutton, C., Llabador, M.A. *et al.* (2021) A missense mutation in IFT74, encoding for an essential component for intraflagellar transport of Tubulin, causes asthenozoospermia and male infertility without clinical signs of Bardet-Biedl syndrome. *Hum Genet*, **140**, 1031-1043.

7 Hammarsjo, A., Pettersson, M., Chitayat, D., Handa, A., Anderlid, B.M., Bartocci, M., Basel, D., Batkovskyte, D., Beleza-Meireles, A., Conner, P. *et al.* (2021) High diagnostic yield in skeletal ciliopathies using massively parallel genome sequencing, structural variant screening and RNA analyses. *J Hum Genet*, **66**, 995-1008.
